# Multilocus sequence typing of diverse phytoplasmas using hybridization probe-based sequence capture provides high resolution strain differentiation

**DOI:** 10.3389/fmicb.2022.959562

**Published:** 2022-09-29

**Authors:** Karolina Pusz-Bochenska, Edel Perez-Lopez, Tyler J. Wist, Harvinder Bennypaul, Daniel Sanderson, Margaret Green, Tim J. Dumonceaux

**Affiliations:** ^1^Agriculture and Agri-Food Canada Saskatoon Research and Development Centre, Saskatoon, SK, Canada; ^2^Department of Biology, University of Saskatchewan, Saskatoon, SK, Canada; ^3^Centre de Recherche et D'innovation sur les Végétaux (CRIV), Faculté des Sciences de L'agriculture et de L'alimentation, Département de Phytologie, Université Laval, Québec, QC, Canada; ^4^Institut de Biologie Intégrative et des Systèmes, Université Laval, Québec, QC, Canada; ^5^Canadian Food Inspection Agency (CFIA), Sidney Laboratory, Centre for Plant Health, North Saanich, BC, Canada; ^6^Department of Veterinary Microbiology, University of Saskatchewan, Saskatoon, SK, Canada

**Keywords:** ‘*Candidatus* Phytoplasma’, phytoplasma taxonomy, hybridization probes, aster yellows, apple proliferation, pear decline, bois noir, X-disease

## Abstract

Phytoplasmas are insect-vectored, difficult-to-culture bacterial pathogens that infect a wide variety of crop and non-crop plants, and are associated with diseases that can lead to significant yield losses in agricultural production worldwide. Phytoplasmas are currently grouped in the provisional genus ‘*Candidatus* Phytoplasma’, which includes 49 *‘Candidatus’* species. Further differentiation of phytoplasmas into ribosomal groups is based on the restriction fragment length polymorphism (RFLP) pattern of the 16S rRNA-encoding operon, with more than 36 ribosomal groups (16Sr) and over 100 subgroups reported. Since disease symptoms on plants are not associated with phytoplasma identity, accurate diagnostics is of critical importance to manage disease associated with these microorganisms. Phytoplasmas are typically detected from plant and insect tissue using PCR-based methods targeting universal taxonomic markers. Although these methods are relatively sensitive, specific and are widely used, they have limitations, since they provide limited resolution of phytoplasma strains, thus necessitating further assessment of biological properties and delaying implementation of mitigation measures. Moreover, the design of PCR primers that can target multiple loci from phytoplasmas that differ at the sequence level can be a significant challenge. To overcome these limitations, a PCR-independent, multilocus sequence typing (MLST) assay to characterize an array of phytoplasmas was developed. Hybridization probe s targeting *cpn60*, *tuf*, *secA*, *secY*, and *nusA* genes, as well as 16S and *rp* operons, were designed and used to enrich DNA extracts from phytoplasma-infected samples for DNA fragments corresponding to these markers prior to Illumina sequencing. This method was tested using different phytoplasmas including ‘*Ca*. P. asteris’ (16SrI-B), ‘*Ca*. P. pruni’ (16SrIII-A),‘*Ca*. P. prunorum’ (16SrX-B), ‘*Ca*. P. pyri’ (16SrX-C), ‘*Ca*. P. mali’ (16SrX-A), and ‘*Ca*. P. solani’ (16SrXII-A). Thousands of reads were obtained for each gene with multiple overlapping fragments, which were assembled to generate full-length (typically >2 kb), high-quality sequences. Phytoplasma groups and subgroups were accurately determined based on 16S ribosomal RNA and *cpn60* gene sequences. Hybridization-based MLST facilitates the enrichment of target genes of phytoplasmas and allows the simultaneous determination of sequences corresponding to seven different markers. In this proof-of-concept study, hybridization-based MLST was demonstrated to be an efficient way to generate data regarding ‘*Ca*. Phytoplasma’ species/strain differentiation.

## Introduction

Phytoplasmas are phytopathogenic bacteria that are grouped into the provisional genus ‘*Candidatus* Phytoplasma’, which includes 49 known species (Bertaccini et al., 2022). These pathogens infect a wide variety of plant species including both crops and weedy species, and can cause agricultural production losses in all production areas of the world. Phytoplasmas are mainly transmitted by hemipteran insects (Weintraub and Beanland, 2006), but they can also be spread through vegetative propagation, grafting, or seeds (Satta et al., 2020; Ranebennur et al., 2022). Phytoplasma-infected plants usually show symptoms such as yellowing, virescence, witches’ broom, phyllody, leaf roll, and generalized decline (Bertaccini, 2022). Symptoms of phytoplasma infection are often overlooked or confused with the response of plants to viral diseases and abiotic stressors; therefore, accurate diagnosis is required for choosing appropriate management strategies. Moreover, like other plant pathogens such as viruses, phytoplasmas are grouped in a variety of groups and subgroups, and genetically distinguishable phytoplasmas can infect the same plant species. Additionally, in many cases, the insect vectors are unknown. Therefore, detecting, monitoring, and controlling diseases associated with these pathogens is very challenging.

Species boundaries for phytoplasmas have been defined by sequence analysis of 16S rRNA-encoding genes, with a sequence identity of the full-length 16S rRNA gene of 98.65% as a cutoff for determining species (Bertaccini et al., 2022). In addition to the ‘*Candidatus*’ species designations, phytoplasmas have been classified into ribosomal groups and subgroups based on RFLP analysis of a fragment of the 16S rRNA gene (Gundersen and Lee, 1996; Zhao et al., 2009). This classification has resulted in the designation of more than 37 ribosomal groups (16Sr) and over 150 subgroups (Wei and Zhao, 2022). Differentiation of phytoplasma strains based on the sequences of 16S rRNA genes is complicated by the relatively low inter-taxon sequence differences observed, and by the fact that the two copies of the 16S rRNA gene within a single genome are in some cases distinct from one another (16S rRNA-encoding gene heterogeneity) (Liefting et al., 1996; Jomantiene et al., 2002; Davis et al., 2003; Perez-Lopez et al., 2019). Therefore, supplementary sequence information from single-copy, protein-coding gene markers is recommended for resolution of phytoplasma strains. There are 49 recognized ‘*Candidatus* Phytoplasma’ species, and while whole-genome draft sequence analysis is becoming available for some phytoplasmas (Firrao et al., 2013; Cho et al., 2020), many of them still do not have genome sequences available.

Multilocus sequence typing (MLST) is well recognized as an improvement over single-marker sequencing for differentiation of bacterial strains in general, and particularly for difficult-to-culture bacteria such as phytoplasmas. Accordingly, MLST has been extensively and recently applied to the differentiation of various groups of phytoplasmas. For example, in defining the taxon ‘*Ca*. P. pruni’, Davis et al. suggested that the strains should best be differentiated by including not only 16S rRNA gene sequences, but also additional sequence information from *secY* and *rp* genes (Davis et al., 2013). Moreover, closely related, but distinct, phytoplasmas belonging to ribosomal group 16SrV (“flavescence dorée”) were successfully differentiated using 16S, *map*, *uvrB-degV*, and *secY* sequences (Arnaud et al., 2007). Strains within group 16SrI associated with azalea little leaf disease were discerned using another set of markers, which included 16S, *rpsS, rplIV, rpsC*, and *secY* genes (Wei et al., 2011). Phytoplasmas classified within ribosomal groups XI and XIV associated with sugarcane white leaf disease, Napier grass stunt, and Bermuda grass white leaf are difficult to differentiate based only on 16S genes, but a MLST scheme using group-specific primers generating a 1 kb fragment of *leuS* (leucyl tRNA synthetase), in combination with *secA* and 16S sequences, provided clarity to their differentiation (Abeysinghe et al., 2016). More recently, MLST has been applied for the differentiation of phytoplasmas within ribosomal group 16SrIV, associated with palm lethal decline. Sequences of the 16S rRNA gene, 16S-23S intergenic spacer region, *secA*, and *groEL* (*cpn60*) demonstrated that three palm lethal decline phytoplasmas could be observed (‘*Ca.* P. palmae’, ‘*Ca*. P. aculeata’, and ‘*Ca*. P. hispanola’), which were distinct from a Tanzanian strain (‘*Ca*. P. cocotanzaniae’). Furthermore, the sequence identity of multiple genes within ribosomal group IV confirmed that distinct ribosomal RNA gene subgroups are properly considered to be the same species, ‘*Ca*. P. aculeata’ (Soto et al., 2021). All of these studies confirm and support the utility of determining the sequences of multiple genetic markers for accurate differentiation of phytoplasma strains.

The objective of the current study was to develop and validate a rapid, convenient, and accurate method of determining the sequences of multiple taxonomic markers for diverse phytoplasma strains. A hybridization-based MLST assay using a set of capture probes corresponding to seven taxonomic markers from a variety of phytoplasmas was developed. Hybridization probes were designed targeting the 16S ribosomal RNA-encoding gene as well as six other protein-coding genes. The protein-coding, single-copy genes selected to develop a multilocus panel were *cpn60* (also known as *groEL* or *hsp60*) (Mitrović et al., 2011; Pérez-López et al., 2016), *tuf* (Marcone et al., 2000), *secA* (Hodgetts et al., 2008), *secY* (Lee et al., 2010), and *nusA* (Shao et al., 2006). In addition, this MLST sequencing panel includes the ribosomal protein (*rp*) operon, which consists of several short genes including *rplV*-*rpsC* and intergenic regions (Martini et al., 2007).

Moreover, to provide a proof-of-concept that the method can detect and type these sequences accurately from a variety of phytoplasmas, six distinct phytoplasmas were examined: ‘*Ca*. P. asteris’ (16SrI), ‘*Ca*. P. pruni’ (16SrIII-A), ‘*Ca*. P. prunorum’ (16SrX-B), ‘*Ca*. P. pyri’ (16SrX-C), ‘*Ca*. P. mali’ (16SrX-A), and ‘*Ca*. P. solani’ (16SrXII-A). The hybridization-based MLST was used to determine the sequences of these markers from each of these phytoplasmas, and in samples with a wide range of phytoplasma concentrations in infected tissues.

## Materials and methods

### Infected plant samples

Plant material indicated in Table 1 that was infected with ‘*Ca.* P. mali’, ‘*Ca.* P. prunorum’, ‘*Ca.* P. pyri’, ‘*Ca.* P. solani’, and ‘*Ca.* P. pruni’ were maintained in appropriate hosts (Table 1) at the Centre for Plant Health, Canadian Food Inspection Agency, North Saanich, BC. Infected canola was collected at the research farm of Agriculture and Agri-Food Canada in Saskatoon, SK. Strain TW1 was collected in 2018 as previously described (Town et al., 2018), and was used to prepare a dilution series of known phytoplasma concentrations in a background of DNA extracted from healthy *B. napus*. Uninfected canola DNA was prepared by germinating phytoplasma-free *B. napus* seeds (Plant Gene Resources of Canada, accession no. CN42942) on filter paper soaked with sterile water, and shoots were collected after 7–10 days of germination in the dark at room temperature. Strain BR1 was collected at the same site as strain TW1, but in 2021. Samples from infected strawberries were collected from fields in Quebec in the summer of 2021 as previously described (Plante et al., 2021). Samples from infected strawberries in Mexican production fields have been described previously (Pérez-López et al., 2017).

**Table 1:** Phytoplasma-infected and uninfected samples used for hybridization-based MLST.

Phytoplasma	Host	Identifier	Group/subgroup	Source location
*‘Ca.* P. asteris’	*Brassica napus*	BnAY-high	16SrI-B	Saskatoon, SK Canada
*‘Ca.* P. asteris’	*Brassica napus*	BnAY-medium	16SrI-B	Saskatoon, SK Canada
*‘Ca.* P. asteris’	*Brassica napus*	BnAY-low	16SrI-B	Saskatoon, SK Canada
*‘Ca.* P. asteris’	*Brassica napus*	BnAY-vlow	16SrI-B	Saskatoon, SK Canada
none	*Brassica napus*	Uninfected	–	Saskatoon, SK Canada
*‘Ca.* P. asteris’	*Brassica napus*	BnAY-BR1	16SrI-B	Saskatoon, SK Canada
*‘Ca.* P. asteris’	*Fragaria × ananassa*	STRAW4	16SrI-R	Quebec, Canada
*‘Ca.* P. asteris’	*Fragaria × ananassa*	Sb7	16SrXIII	Jalisco, Mexico
*‘Ca.* P. asteris’	*Fragaria × ananassa*	Sb41	16SrXIII/16SrI	Jalisco, Mexico
*‘Ca.* P. prunorum’ (ESFY)	*Prunus marianna*	2813-1C1	16SrX-B	Sidney, BC, Canada
*‘Ca.* P. mali’ (AP)	*Malus domestica*	3516-1A1	16SrX-A	Sidney, BC, Canada
*‘Ca.* P. pyri’ (PD)	*Pyrus communis*	1847-2C1	16SrX-C	Sidney, BC, Canada
*‘Ca.* P. pyri’ (PYLR)	*Prunus persica*	1847-4C3	16SrX-C	Sidney, BC, Canada
*‘Ca.* P. pruni’	*Syringa ‘Charisma’*	3194-2A1	16SrIII-A	Sidney, BC, Canada
*‘Ca.* P. pruni’	*Prunus avium*	1847-6A1	16SrIII-A	Sidney, BC, Canada
*‘Ca.* P. solani’	*Vitis vinifera^4^*	NA18-199	16SrXII-A	Sidney, BC, Canada
none	*Vitis vinifera*	Uninfected	–	Sidney, BC, Canada

### DNA extraction and quality control

Midribs were excised from the leaves of infected plants for all samples except TW1 (inflorescence) and healthy canola (shoots from germinated seeds). The tissue (~0.1 g) was cut into ~5 mm pieces, placed into a 2 ml tube with 2 sterile steel beads (3.2 mm, BioSpec Products), and immediately frozen in liquid nitrogen. Frozen tissue was pulverized using a homogenizer (Retsch, model no. MM 400) using 2 pulses of 30 s of shaking at 30 Hz. Powdered samples were then briefly centrifuged, and DNA was extracted using a Qiagen Plant DNA mini kit. DNA was eluted into 100 μl of 10 mM Tris-Cl pH 8.0 (kit elution buffer). DNA concentration was measured using a Qubit Broad Range kit (Invitrogen).

Quantitative PCR (qPCR) was used to determine the level of phytoplasma in each of the DNA extracts from infected tissue samples prior to hybridization and sequencing. Primers and probes were purchased from IDT (Coralville, IA). Their sequences, along with amplification conditions, are provided as supplementary information (Supplementary Table S1). For all samples except ‘*Ca.* P. pruni’, qPCR used 1x SsoFast Universal Probes Supermix (Bio-Rad), 0.3 μM each primer, and 0.2 μM probe in a final volume of 20 μl. For ‘*Ca.* P. pruni’, qPCR used 1x SsoFast Universal Probes Supermix, 0.4 μM primer 16SF, 1.2 μM primer 16SR, 0.2 μM probe 16S72, and 1x SsoFast Universal Probes Supermix. qPCR standards were prepared from plasmid DNA containing the *cpn60* universal target sequence from the respective phytoplasma (Muirhead et al., 2019a). qPCR standards for ‘*Ca.* P. pruni’ were prepared using infected cherry DNA calibrated to 200,000 copies/μl, and infected plum DNA at 200 copies/μl. Amplifications used a C1000 thermocycler base with a CFX96 qPCR module and results were analyzed using Bio-Rad CFX Manager v3.1.1517.0823. Statistical correlations between *cpn60* input DNA copies measured by qPCR and *cpn60* read numbers post-hybridization were calculated using SigmaPlot v14.5.

To prepare a dilution series of ‘*Ca.* P. asteris’, DNA extracted from infected *B. napus* inflorescence (strain TW1) was mixed with DNA extracted from uninfected *B. napus* shoots. Briefly, AY *cpn60* copies were measured in the TW1 inflorescence extract using qPCR, then the sample was adjusted to approximately 5×10^5^ *cpn60* copies/ul in 10 mM Tris-Cl, pH 8.0. This sample is referred to as BnAY-high. Three serial 10-fold dilutions of BnAY-high were then prepared, with 50 μl of DNA from uninfected canola (Bn-H) added to each dilution and the volume adjusted to 110 μl using 10 mM Tris-Cl, pH 8.0. These “spiked-in” samples are labeled BnAY-medium, BnAY-low, and BnAY-vlow (very low). The DNA concentration and number of AY *cpn60* copies/μl were measured in each final pool as described.

### PCR amplification and cloning of target gene sequences

The 16S rRNA-encoding gene locus was amplified from DNA extracts using a nested PCR strategy consisting of primers P1 (Deng and Hiruki, 1991) and P7 (Schneider et al., 1995) in a first round, generating a product of >1.8 kb. This PCR product was diluted 1:30, then 2 μl was used as template in a secondary PCR step using primers R16F2n and R16R2 (Gundersen and Lee, 1996), which generated the ~1.2 kb F2nR2 amplicon that is commonly used for phytoplasma detection and typing(Zhao et al., 2009). Both rounds of PCR for 16S used 1x PCR buffer (Invitrogen), 2.5 mM MgCl_2_, 500 nM dNTPs, and 400 nM each primer using previously described methodologies (Pérez-López et al., 2017). Thermal cycling conditions were 95°C, 10 min (1x), 95°C, 1 min; 55°C, 1 min; 72°C, 1.75 min (35X), 72°C, 10 min (1x). The universal target region from the *cpn60* gene (Dumonceaux et al., 2014) was amplified using a primer cocktail as described at cpnclassiphyr.ca (Muirhead et al., 2019a). Amplicons were generated from the *rp* operon of AY infected samples using nested PCR with primers rpF1 and rpR1 (Lim and Sears, 1992) in the first round, and rp(I)F1A/rp(I)R1A in the second round as described (Lee et al., 2004).

Amplicons were cloned into the vector pGEM-T Easy (Promega, Madison, WI) according to the instructions provided, then plasmids were transformed into chemically competent *E. coli* TOP10 cells (Life Technologies). 5–6 individual clones were sequenced from each amplicon using a commercial sequencing service (Eurofins Genomics, Toronto, ON).

### Hybridization probe design

Gene targets for probe design were selected from publicly available sequences at GenBank (Table 2). 27 genes were selected from genome sequences and individual gene sequences, with 7 targets from each of ‘*Ca.* P. asteris’ (16SrI-B); ‘*Ca.* P. mali’ (16SrX-A); ‘*Ca.* P. solani’ (16SrXII-A), and 6 genes from ‘*Ca.* P. pruni’ (16SrIII-A). A total of 351 probes were designed with a length of 120 nucleotides and 1x tiling density using the IDT X-gen design tool found.^1^ Probe sequences are provided as Supplementary File S1.

**Table 2:** GenBank accession numbers of gene sequences used for probe design.

Gene target	16Sr group/subgroup			
	16SrI-B	16SrIII-A	16SrX-A	16SrXII-A
*cpn60*	QGKT01000000	NA^1^	NC_011047	FO393427
*tuf*	QGKT01000000	NZ_LHCF01000000	NC_011047	FO393427
*secY*	KP796188^2^	NZ_LHCF01000000	NC_011047	FO393427
*secA*	QGKT01000000	NZ_LHCF01000000	NC_011047	FO393427
*nusA*	QGKT01000000	NZ_LHCF01000000	NC_011047	FO393427
16S-23S	KX551964	HQ589202	X68375	JQ730740
*rp*	QGKT01000000	JQ360955^3^	EF193367	KC481241
Total nucleotides	10687	9162	10518	10546
Total number of probes	92	78	90	91
Probe length	120	120	120	120
Tiling density	1x	1x	1x	1x

^1^*‘Ca.* P. pruni’ (16SrIII) lacks a *cpn60* gene (Saccardo et al., 2012).

^2^*secY* annotated in TW1 genome appears to have a premature stop codon.

^3^GenBank entry for strain CX rp locus had N’s – therefore changed to a clean sequence from related strain CX-95.

### Hybridization and sequencing

Detailed protocols for DNA preparation for hybridization and sequencing have been published elsewhere (Dumonceaux et al., 2017), and these were generally followed. Wherever possible, DNA was diluted to 2.5 ng/ul in a total volume of 100 μl of 10 mM Tris-Cl pH 8.0. If the DNA concentration was below this level, it was used at its extracted concentration, with the volume adjusted to 100 μl for shearing. Shearing proceeded using a Bioruptor 300 (Diagenode # B01020001) in 0.2 ml shearing tubes (Diagenode #C30010015) on a setting of “high” with 30 cycles of 30 s on, 45 s off, and cooling to 4°C. Sheared DNA was concentrated using Amicon YM-30 filter membranes to a volume of 60 μl. DNA concentration was determined using a Qubit Broad Range kit (1 μl). Illumina adaptors and indices were added using a NEBNext Ultra DNA library prep kit for Illumina (NEB, cat no E7370) according to the manufacturer’s instructions. Prior to index addition, size selection was used to isolate fragments of 400–500 bp by using 35 μl of SPRI beads (Cytiva) in the first bead selection and 25 μl in the second bead selection. Index addition proceeded using 8 cycles of PCR under the conditions recommended by the manufacturer, and indexed fragments were purified using a 1:1 volume ratio of SPRI beads (Cytiva) and eluted in a volume of 30 μl. Results were examined using a TapeStation (Agilent) and DNA concentration was determined using a Qubit High Sensitivity (HS) kit.

Hybridization was performed using an xGen hybridization and wash kit (IDT) and the recommended protocols (IDT xGen hybridization capture of DNA libraries manual version 4, 2019). Up to 500 ng of indexed DNA was added to each hybridization reaction along with xGen universal blockers (TS mix, IDT). All samples were hybridized with the same set of capture probes containing sequences from all seven markers from all of the targeted phytoplasmas (Table 1). Hybridizations proceeded at 65°C for 16 h. After washing, libraries were purified using a 1:1 volume ratio of SPRI beads (Cytiva) and amplified for 14 cycles using the x-Gen library amplification primer (IDT) and KAPA HiFi HotStart ReadyMix (Roche) under cycling conditions recommended by the manufacturer. After another SPRI purification, DNA concentration was determined using a Qubit HS kit. If the DNA yield was insufficient (under approx. 1 ng/ul), a second amplification reaction was performed using 1 μl of the first amplification reaction with 19 μl water and the same primer; products were then re-purified using SPRI beads. Samples were pooled to 4 nM and sequenced using an Illumina nano kit v2 (500 cycles) using a MiSeq instrument according to the manufacturer’s recommendations.

### Data analysis

Illumina reads were processed with trimmomatic (v0.39) to remove adapters and low quality bases (<Q15) and R1/R2 reads were merged using flash2 (v2.2.00). Merged reads were mapped to the sequences of the genes used for hybridization probe design using bowtie2 --local (v2.4.4). The reference sequences used for mapping are included as Supplementary File S2. Reads mapped to each target sequence were extracted using samtools (v1.9), and the corresponding fastq files were then assembled as individual bins using transabyss (v2.0.1). Assemblies were filtered to include only contigs >500 nucleotides.

To remove off-target reads that mapped to undesired 16S sequences (16S genes from other bacteria, along with host chloroplast and mitochondrial genes), assembled contigs that were not phytoplasma 16S sequences were identified using BLAST. These contigs were then added as reference sequences for a second round of mapping for 16S-mapped reads only using bowtie2 --local to remove these non-target sequences from the assembly dataset. This was done only in cases where the initial assembly using the mapped 16S genes generated short or only nonspecific contigs. The code used for mapping and assembly is provided as Supplementary File S3.

### Phylogenetic analysis

Sequences assembled from the post-hybridization sequencing and mapping data were oriented manually by BLAST to determine sequence orientation (coding, or reverse-complement), then reverse-complementing as necessary. Oriented sequences were aligned using clustalw, then trimmed manually to the length of the shortest sequence in the alignment. Phylogenetic relationships among the sequences were inferred by using the Neighbor Joining algorithm (Saitou and Nei, 1987) in MEGA X (Kumar et al., 2018). Trees were bootstrapped using 1,000 replications, and consensus trees were reported with percentages of trees in which the associated taxa clustered together indicated next to each branch. The evolutionary distances were computed using the Maximum Composite Likelihood method (Tamura et al., 2004).

**Figure 1:**
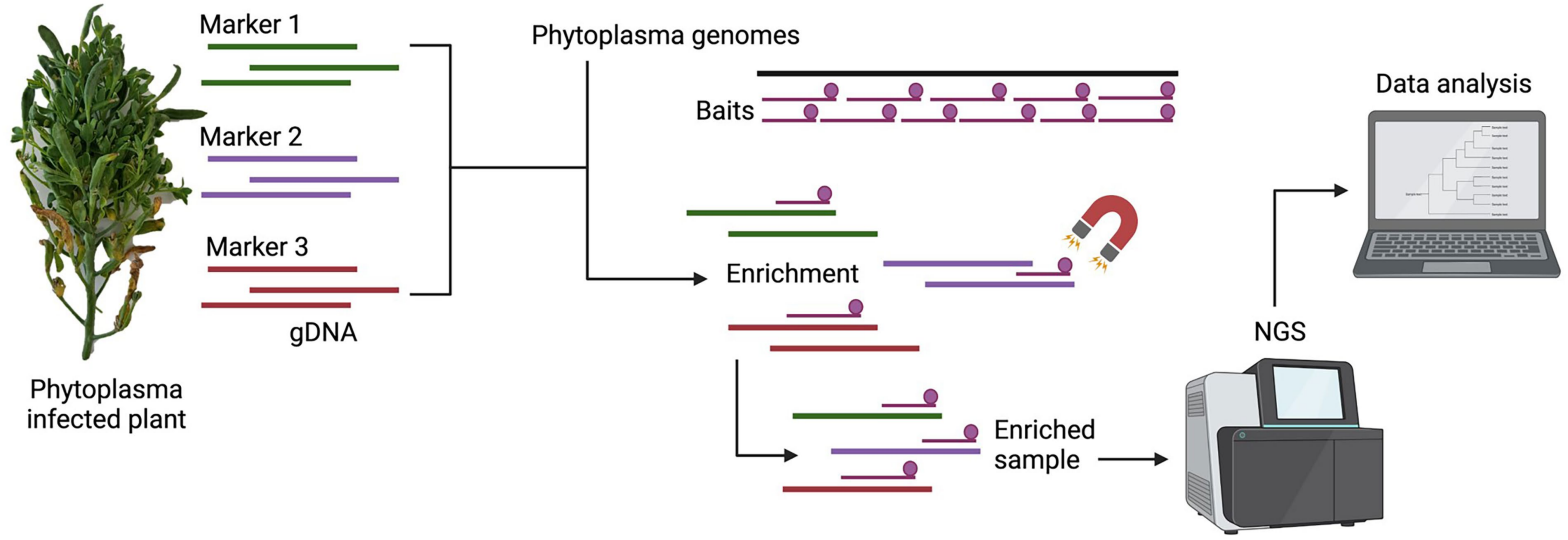
Schematic diagram of the hybridization-based MLST method.

A schematic representation of the methodology used in this study is presented in Figure 1.

## Results

### DNA extraction and qPCR

DNA yield from infected leaf midribs was somewhat variable, ranging from under 1 ng/ul (‘*Ca.* P. solani’, Sb41) to approximately 15 ng/ul in lilac infected with ‘*Ca.* P. pruni’ (Table 3). The mean DNA concentration for all samples was 8.74 ng/ul. Similarly, samples displayed a wide range of phytoplasma levels in the infected plant tissue; C_q_ values were quite high in some samples (25–27 for ESFY, AP, and PD) but lower in other samples approximately 20 in the ‘*Ca.* P. pruni’ and ‘*Ca.* P. solani’-infected samples (Table 3). The sample from the canola plant infected with ‘*Ca.* P. asteris’ strain BR1 had the lowest C_q_ (14) of the samples examined, reflecting the highest phytoplasma concentration. Samples of canola DNA that were purposefully constructed to contain various levels of phytoplasma DNA had C_q_ values ranging from 17.62 (BnAY-TW1) (‘*Ca.* P. asteris’-high) to 27.12 (BnAY-TW1-vlow). All samples were successfully sheared and indexed, regardless of the DNA input amounts.

**Table 3:** DNA yield and phytoplasma levels in each sample.

Phytoplasma	Host	strain ID/CPH no	Group/subgroup	(DNA), ng/μl	qPCR C_q_ mean	qPCR copies/μl
BnAY-high	*Brassica napus*	TW1	16SrI-B	16.1	17.62	566875
BnAY-medium	*Brassica napus*	TW1	16SrI-B	9.76	20.29	88627
BnAY-low	*Brassica napus*	TW1	16SrI-B	9.53	23.66	8524
BnAY-vlow	*Brassica napus*	TW1	16SrI-B	9.45	27.12	765
None	*Brassica napus*	uninfected	–	18.8	0	0
‘*Ca.* P. asteris’	*Brassica napus*	BR1	16SrI-B	4.7	14.03	8395731
‘*Ca.* P. asteris’	*Fragaria × ananassa*	STRAW4	16SrI-R	3.77	26.79	531
‘*Ca.* P. asteris’; ‘*Ca.* P. hispanicum’	*Fragaria × ananassa*	Sb7^1^	16SrXIII	1.99	21.68 (XIII)/ND^3^ (I)	34836 (XIII)/ND (I)
‘*Ca.* P. asteris’; ‘*Ca.* P. hispanicum’	*Fragaria × ananassa*	Sb41^2^	16SrXIII/16SrI	too low	32.49 (XIII)/ 28.62 (I)	8.7 (XIII)/1,024 (I)
‘*Ca.* P. prunorum’ (ESFY)	*Prunus marianna*	2813-1C1	16SrX-B	6.8	27.05	1740
‘*Ca.* P. mali’ (AP)	*Malus domestica*	3516-1A1	16SrX-A	12.3	25.22	4292
‘*Ca.* P. pyri’ (PD)	*Pyrus communis*	1847-2C1	16SrX-C	12.4	27.04	500
‘*Ca.* P. pyri’ (PYLR)	*Prunus persica*	1847-4C3	16SrX-C	7.9	22.53	9531
‘*Ca.* P. pruni’	*Syringa ‘Charisma’*	3194-2A1	16SrIII-A	14.9	19.66	216177
‘*Ca.* P. pruni’	*Prunus avium*	1847-6A1	16SrIII-A	9.6	19.29	256025
‘*Ca.* P. solani’	*Vitis vinifera^4^*	NA18-199	16SrXII-A	0.756	20.46	558826
None	*Vitis vinifera*	uninfected	–	1.13	0	0

^1^Sample was previously determined to be infected with ‘*Ca.* P. hispanicum’ (16SrXIII) – (Pérez-López et al., 2017), and was not previously known to be co-infected with ‘*Ca.* P. asteris’.

^2^Sample was previously determined to be co-infected with ‘*Ca.* P. hispanicum’ (16SrIII) and 16SrI (‘*Ca.* P. asteris’) – (Pérez-López et al., 2017).

^3^ND, not determined.

### Hybridization and Illumina sequencing

Post-hybridization read numbers mapping to each gene are shown in Table 4. Depending on the amount of phytoplasma DNA in the sample, the configuration of the sequencing run, and other technical variables, hundreds to thousands of reads typically mapped to each gene. Fewer reads were observed in the lower concentration samples such as ESFY and PD, with the latter showing one of the lowest proportions of reads mapping at ~3% (Table 4). In most cases, the 16S ribosomal gene had the highest number of mapped reads. Samples from uninfected canola and grape showed lower numbers of mapped reads compared to the corresponding infected plants, especially for the protein-coding genes (Table 4). Without hybridization, samples from the canola phytoplasma mixtures produced very few reads mapping to the target genes (Table 5).

**Table 4:** Number of sequencing reads mapping to each gene after hybridization.

Phytoplasma^1^	strain ID/CPH no	*cpn60*	16S-23S	*tuf*	*secY*	*secA*	*nusA*	*rp*
BnAY-high	TW1	4732	8402	3482	3298	6873	2970	3208
BnAY-medium	TW1	6299	11399	4334	4358	9034	3935	4097
BnAY-low	TW1	293	1134	309	163	449	172	203
BnAY-vlow	TW1	71	285	53	53	86	37	55
None	uninfected	0	192	2	0	1	2	0
‘*Ca.* P. asteris’	BR1	13214	23511	9714	9075	18126	8113	9099
‘*Ca.* P. asteris’	STRAW4	12090	19426	8011	6821	10024	6003	7780
‘*Ca.* P. asteris’	Sb7	250	233716	1341	149	285	128	183
‘*Ca.* P. asteris’	Sb41	25891	90732	22429	18064	34831	16452	22576
‘*Ca.* P. prunorum’ (ESFY)	2813-1C1	840	5000	986	755	1094	113	1704
‘*Ca.* P. mali’ (AP)	3516-1A1	13714	9917	8302	4767	12750	6900	8314
‘*Ca.* P. pyri’ (PD)	1847-2C1	460	729	389	53	253	107	412
‘*Ca.* P. pyri’ (PYLR)	1847-4C3	5671	10033	4320	188	3028	1579	5015
‘*Ca.* P. pruni’	3194-2A1	–	6225	3532	3888	7381	3384	4384
‘*Ca.* P. pruni’	1847-6A1	–	6150	5534	8692	19253	8184	8935
‘*Ca.* P. solani’	NA18-199	11281	23101	7006	10328	20863	8109	11152
None	uninfected	86	3784	46	41	76	57	14

^1^The same set of 351 hybridization probes, containing capture probes for all genes from all of the targeted phytoplasmas, was used in each hybridization.

**Table 5:** Number of reads mapping to each gene in non-hybridized, spiked samples.

Phytoplasma	*cpn60*	16S-23S	*tuf*	*secY*	*secA*	*nusA*	*rp*
BnAY-high	4	17	3	0	3	4	2
BnAY-medium	3	45	2	1	2	0	3
BnAY-low	0	122	0	1	1	0	1
BnAY-vlow	26	268	14	15	27	12	23

The qPCR assays targeting the *cpn60* gene (Supplementary Table S1) provided input concentrations of phytoplasma. Examining the relationship between the initial phytoplasma levels in each sample and the number of reads mapping to *cpn60* generated revealed a significant correlation (Pearson *r*^2^ = 0.696, *p* < 0.05; Spearman’s ρ = 0.770, *p* < 0.05), suggesting that the number of reads mapping was related to the starting concentration of phytoplasma in the sample, at least within the same Illumina run. Such a determination could not be made for the other protein-coding genes due to the lack of gene-targeted qPCR assays, but this correlation was not evident in the 16S ribosomal gene datasets (Table 4). The actual number of reads mapping to each target was variable across samples and was quite high in some samples, even those that had relatively low concentration of phytoplasma such as STRAW4 (Table 4).

A large number of reads typically mapped to the 16S ribosomal gene target in all samples, including uninfected tissue. In fact, the large number of reads mapping to the 16S ribosomal gene in each sample were found to include many reads that did not correspond to the target gene, including host chloroplast and mitochondrial genes, 16S ribosomal genes from non-phytoplasma bacteria, and other nonspecific DNA sequences. Examination of the nucleotide identities of the post-hybridization reads that mapped to the 16S ribosomal gene compared to the probe sequences using BLAST revealed that the lower spike levels contained a substantial proportion of reads with <95% nucleotide identity to any of the probes (Supplementary Figure S1A). These non-target reads interfered in some samples with the assembly process, to the extent that the ‘*Ca.* P. asteris’ samples spiked at low and very low levels assembled only nonspecific host chloroplast sequences (Supplementary Table S2). Other samples, such as PYLR, assembled only 16S contigs that were too short for classification using the *i*PhyClassifier (Supplementary Table S2). Incorporating a second mapping step that included these nonspecific assembled sequences as mapping targets provided a much higher proportion of reads (average read length 274 bp) with >98% identity to a probe sequence (Supplementary Figure S1B), and greatly improved most of the 16S assemblies (Supplementary Table S2). This was most pronounced with the ‘*Ca.* P. asteris’ mixtures, with the medium and low titer samples yielding 16S assemblies that were > 2 kb after this step, and the very low sample yielding a 925 nucleotide 16S assembly compared to no detectable 16S sequence after the initial mapping (Supplementary Table S2). This two-step mapping strategy for 16S rRNA sequences was not required for other samples. In particular, the 16SrIII samples generated 16S assemblies >2.4 kb by mapping only to the target 16S gene sequence, so in this case a two-step mapping strategy was not necessary (Supplementary Table S2). In contrast, the initially mapped reads from the spiked canola samples corresponding to a protein-coding gene (*cpn60*) had >98% sequence identity to a probe sequence at all spike levels, and therefore represented high-quality sequence reads that were used in the assemblies (Supplementary Figure S1C).

Assembly of the mapped reads for each gene generated sequences that ranged from 599 nucleotides (PYLR *secY*) to 3,225 nucleotides (‘*Ca.* P. pruni’ 6A1 *secA*) (Table 6). Most of the sequences were at least 1,500 nucleotides, with an average contig length of 1927 nucleotides and a median of 1867 nucleotides. The *secA* assemblies were the longest (average of 2,812 nucleotides), while the *nusA* assemblies tended to be shorter (average of 1,438 nucleotides). This is consistent with the target gene lengths (*secA* is the longest target gene at ~2,500 nucleotides, and *nusA* the shortest at ~1,075 nucleotides).

**Table 6:** Longest assembled contig lengths (in base pairs) and RFLP-based typing results (16S and *cpn60* only) for each gene in each sample.

Sample	*cpn60*	*cpn60* RFLP type^1^	16S-23S	16Sr RFLP subgroup^2^	*tuf*	*secY*	*secA*	*nusA*	*rp*
BnAY-high	2367	I-IB (1.0)	2654	I-B (1.0)	1855	1963	3173	1813	1993
BnAY-medium	2306	I-IB (1.0)	2557	I-B (1.0)	1878	1867	3177	1784	1899
BnAY-low	1906	I-IB (1.0)	2230	I-B (0.97)	1494	1561	2650	1255	1628
BnAY-vlow	1926	I-IB (1.0)	925	Not typeable^3^	1494	1490	2781	1217	1714
BnAY-BR1	2131	I-IB (1.0)	2396	I-B (1.0)	1697	1853	2903	1572	1763
‘*Ca.* P. asteris’ – STRAW4	2216	I-IC (1.0)	2561	I-R (0.98)	1843	1879	3056	1649	1801
‘*Ca.* P. asteris’ – Sb7	1258	I-IIIB (1.0)	2631	XIII-H (0.98)	1007	1432	2732	1189	1411
‘*Ca.* P. asteris’ – Sb41	2200	I-IIIB (1.0)	2463	I-B (0.97)	1793	1808	2970	1611	1787
‘*Ca.* P. prunorum’ (ESFY)	1749	X-IF (1.0)	2184	X-B (0.98)	1299	945	2130	620	1638
‘*Ca.* P. mali’ (AP)	2330	X-IA (1.0)	2287	X-A (1.0)	1943	1805	2975	1836	1854
‘*Ca.* P. pyri’ (PD)	1876	X-IC (1.0)	2085	X-C (1.0)	1496	687	1970	752	1622
‘*Ca.* P. pyri’ (PYLR)	1972	X-IC (1.0)	2218	X-C (0.99)	1766	599	2143	1125	1736
‘*Ca.* P. pruni’ – 2A1	No data^4^	No data	2435	III-A (1.0)	1861	1915	3214	1755	2028
‘*Ca.* P. pruni’ – 6A1	No data^4^	No data	2400	III-A (1.0)	1912	1917	3225	1785	2046
‘*Ca.* P. solani’ – NA18-199	2147	XII-IA (1.0)	1950	XII-A (1.0)	1638	994	3082	1611	1767

^1^*cpn60* RFLP typing results determined using the CpnClassiPhyr (Muirhead et al., 2019a). *F*-value is provided in parentheses.

^2^16S RFLP typing results determined using the *i*Phyclassifier (Zhao et al., 2013). *F*-value is provided in parentheses.

^3^Assembled 16S rRNA-encoding gene was too short for *i*Phyclassifer (<1,200 bp).

^4^*‘Ca.* P. pruni’ (16SrIII) does not contain *cpn60* (Saccardo et al., 2012).

### Sequence typing and phylogenetic analysis

#### 16S ribosomal RNA gene and *cpn60* sequence types

All samples yielded 16S ribosomal RNA gene and *cpn60* sequences that typed correctly using the corresponding RFLP-based classifiers (Table 6). In all cases except Sb7 (Strawberry – Mexico) and STRAW4 (Strawberry-Quebec), the *cpn60* and 16S sequence types were in agreement. For Sb7, the *cpn60* sequence generated typed as *cpn60* I-IIIB: maize bushy stunt (MBS), while the 16S sequence corresponded to the group 16SrXIII-H. For STRAW4, the *cpn60* typed as I-IC while the 16S sequence typed as 16SrI-R (Table 6).

#### Phylogenetic analysis of target gene assemblies

##### 16S rRNA gene sequences

**Figure 2:**
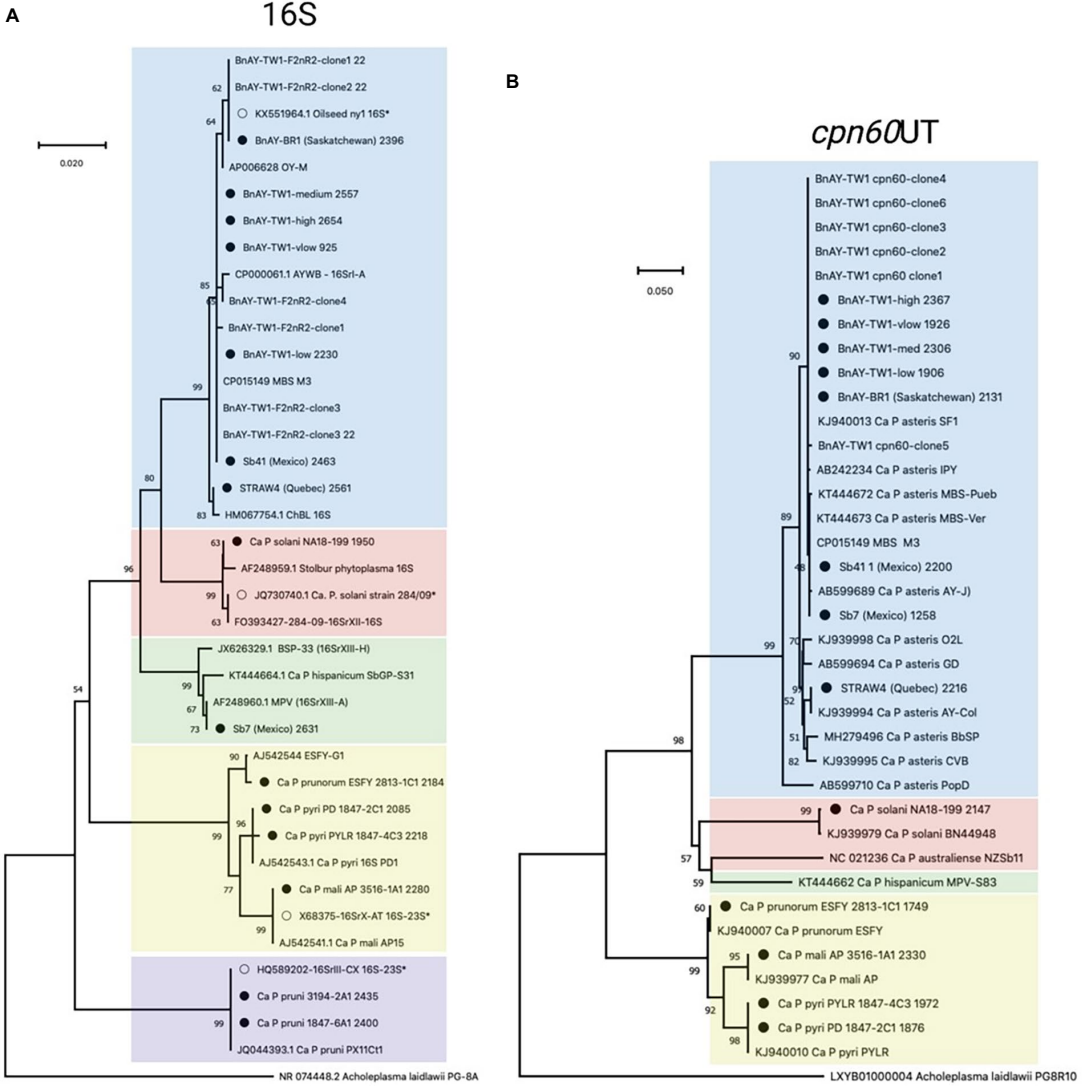
Phylogenetic analysis of 16S **(A)** and *cpn60* UT **(B)** sequences generated in this study. 16S sequences corresponded to the F2nR2 fragment (~1.2 kb), and *cpn60* sequences were trimmed to the universal target length (~550 bp) using the CpnClassiPhyR (Muirhead et al., 2019a). Phylogenetic analysis was performed using the Neighbor Joining algorithm using 1000 replicates, as described in Methods. Sequences corresponding to the samples analyzed in this study (Table 1) are indicated with a filled circle, while samples with an open circle represent the sequences used for hybridization probe design (Table 2). Samples are color-coded according to the 16Sr groups represented within the samples and the probes described in this study – 16SrI (blue); 16SrXII (pink); 16XrXIII (green); 16SrX (yellow); 16SrIII (purple; 16S only).

16S rRNA-encoding gene sequences generated from the samples that were infected with group 16SrX phytoplasmas (AP, ESFY, PYLR, PD) clustered with their respective reference sequences (Figure 2A), in most cases with nearly zero branch length, indicating sequence identity. Although the hybridization probes were designed using the 16S rRNA-encoding gene sequence of 16SrX-A (strain AT; Table 1), hybridization of other subgroups 16SrX-C (PYLR/PD) and 16SrX-B (ESFY) was also successful. Similarly, the 16S ribosomal RNA gene sequences generated from samples infected with 16SrIII clustered with their respective reference sequences with near sequence identity (Figure 2A). The grape sample infected with 16SrXII was slightly different from the strain used for hybridization probe design, and clustered with a “stolbur” phytoplasma strain within the same subgroup 16SrXII-A.

The samples that were infected with various 16SrI subgroups generated 16S rRNA gene sequences that were generally consistent with the expected groupings. The AY-infected strawberry from Quebec (STRAW4) yielded a sequence that clustered with a strain of 16SrI-R (Figure 2A), consistent with the RFLP-based typing results (Table 6). The AY-infected canola samples from Saskatchewan provided 16S gene sequences that mostly clustered with strains from subgroup 16SrI-B, as expected from the RFLP typing results. This included strain BnAY-BR1, which was collected in 2021 from the same field as strain BnAY-TW1 (collected in 2017). Examination of F2nR2 clone sequences generated from strain BnAY-TW1 revealed that 5/6 clones clustered with 16SrI-B, while one (clone 4) was more closely related to 16SrI-A. The clones that clustered within 16SrI-B were therefore differentiated into two types, consistent with the RFLP typing results for these clones. The sequences that were assembled from the hybridization, however, all appeared to cluster with the 16SrI-B sequences. These sequences were slightly distinct from the sequence used for the design of hybridization probes (Figure 2A).

Strawberry samples Sb41 and Sb7, which were collected in Jalisco, Mexico (Pérez-López et al., 2017), provided 16S gene sequences that clustered with 16SrI-B (Sb41) and 16SrXIII-A (Sb7) (Figure 2A). Despite the previously reported double infection of sample Sb41 with 16SrXIII and 16SrI (Pérez-López et al., 2017), no evidence of 16S rRNA gene sequences from 16SrXIII was found in sample Sb41.

##### cpn60

The length of the *cpn60* assemblies (Table 6) allowed the use of more than the ~550 bp universal target region (Muirhead et al., 2019b) for phylogenetic analysis. The sample infected with 16SrX-A generated a *cpn60* sequence that was identical to that of the strain that was used for hybridization probe design (Supplementary Figure S2). There is no full-length *cpn60* sequence available for ESFY (16SrX-B), but the PYLR/PD (16SrX-C) *cpn60* sequences were identical to a strain from the GenBank database (Supplementary Figure S2), indicating that the assemblies were correct for the 16SrX-C samples. In addition, the *cpn60* sequence from the 16SrXII-infected sample clustered with the corresponding sequence used for hybridization probe design (Supplementary Figure S2).

Sequences generated from infected canola plants in Saskatchewan, including BnAY-TW1 and BnAY-BR1, clustered with the sequence used to generate the hybridization probes with zero branch length (Supplementary Figure S2). However, the sequences from infected strawberries clustered independently with no reference sequence (STRAW4, Quebec), or with subgroup 16SrI-B detected in samples Sb7 and Sb41 from Mexico.

**Figure 3:**
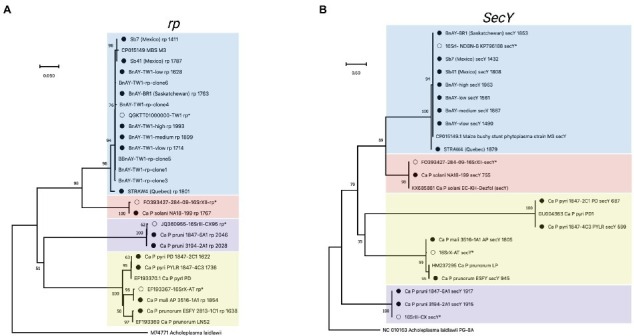
Phylogenetic analysis of *rp* **(A)**, and **(B)** *secY* sequences generated in this study. Sequences were trimmed manually to the length of the shortest sequence analyzed (see Methods). Samples are indicated as described for Figure 2, and the length of the original assembly prior to trimming (Table 6) is indicated for each sample (filled circles).

Trimming the sequences to the universal target using the CpnClassiPhyr (Muirhead et al., 2019a) permitted the use of an expanded set of reference sequences, along with RFLP analysis. This trimming provided results that were consistent with the longer sequences (Figure 3A), and further showed that the 16SrX samples corresponding to ESFY, PYLR, and PD clustered with their respective reference sequences, and that the sample from infected strawberries in Quebec (STRAW4) clustered with the *cpn60* UT I-IC sample (AY-Col). Moreover, all six *cpn60* UT amplicon clones generated from strain BnAY-TW1 were identical to the assembled sequence and to the reference sequence used to design the hybridization probes (Figure 2B).

##### rp

Results of phylogenetic analysis using the *rp* sequences assembled from hybridized infected samples are shown in Figure 3A. A similar theme was observed; sequences obtained from infected ‘*Ca.* P. mali’ clustered with the sequence used to generate the probes, while the sequences from the other 16SrX subgroups represented by strains ESFY, PYLR, and PD clustered separately, and were identical to their respective reference sequences. The samples infected with 16SrIII and 16SrXII also generated sequences that were identical to the sequence used for hybridization probe design. The AY infected canola samples were all virtually identical to one another and to the probe sequence, along with 5 PCR-amplified *rp* clones from BnAY-TW1. The infected strawberry from Quebec generated a distinct sequence, while the sequences from infected strawberries in Mexico clustered with the aster yellows *rp* sequences.

##### secY

Despite the fact that the *secY* reads generated assemblies that were, on average across all strains analyzed, longer (average length 1,514 bp – Table 6) than those of the shorter target gene *nusA* (average length 1,438 bp – Table 6), the alignment and trimming of the *secY* sequences to a common fragment resulted in the shortest sequence comparison among the genes analyzed; phylogenetic analysis was based on a fragment of only ~530 nucleotides. This was mostly due to the shorter assemblies that were generated for ‘*Ca.* P. prunorum’ (ESFY) and ‘*Ca.* P. pyri’ (PD and PYLR) – see Table 6. The *secY* sequences assembled from infected samples showed that this gene is highly discriminatory for 16SrX strains (Figure 3B). The >1,800 nucleotide sequence that was assembled from the ‘*Ca.* P. mali’-infected sample, as expected, was almost identical to the sequence used for probe design, while the ESFY (16SrX-B) sequence was identical to its reference sequence and distinct from the ‘*Ca.* P. mali’ (16SrX-A) sequence. However, the *secY* sequences that were assembled from ‘*Ca.* P. pyri’ (16SrX-C) displayed a long branch length compared to the other 16SrX samples, consistent with their lower nucleotide sequence identity (~90%). As with other genes, the *secY* sequences generated from 16SrIII samples were identical to the target sequence, and the sequence assembled from the ‘*Ca.* P. solani’-infected sample was nearly identical to its target sequence. *secY* sequences were less discriminatory with the 16SrI samples, as all sequences except STRAW4 were identical, including the closely related samples generated from the infected strawberries in Mexico that were also identical to MBS *secY* (Figure 3B).

##### secA

**Figure 4:**
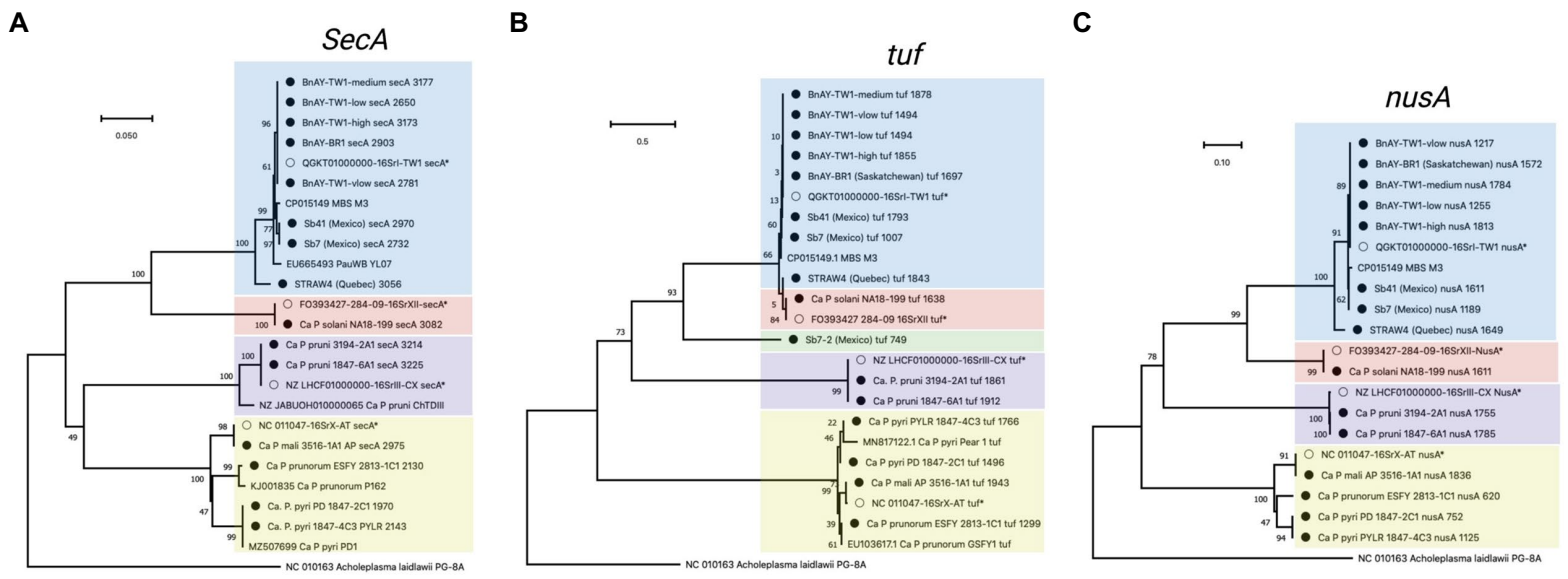
Phylogenetic analysis of *secA* **(A)**, *tuf* **(B)**, and *nusA* **(C)** sequences generated in this study. Sequences were trimmed manually to the length of the shortest sequence analyzed (see Methods). Samples are indicated as described for Figure 2, and the length of the original assembly prior to trimming (Table 6) is indicated for each sample (filled circles).

The longest target gene in the MLST scheme was *secA*, which was approximately 2,500 nucleotides. Phylogenetic analysis was therefore based on a relatively long fragment of approximately 1,970 nucleotides, corresponding to the length of the shortest sequence generated (‘*Ca.* P. pyri’ PD). The sequence generated for ‘*Ca.* P. mali’ was identical to the sequence used for group 16SrX probe design, while the sequences obtained from the other 16SrX-infected samples clustered separately (Figure 4A). While there was no reference sequence available for ESFY, the PYLR and PD sequences were identical to a reference sequence from ‘*Ca.* P. pyri’ (isolate PD1; Figure 4A). Similarly, the 16SrXII- and 16SrIII-infected samples generated *secA* sequences that were identical to the sequence used for probe design (Figure 4). The *secA* sequences of the infected strawberries from Mexico clustered with MBS phytoplasma (16SrI-B) *secA* sequence, although the sequence differences were not as great as those observed with other markers. The infected strawberry sample from Quebec (STRAW4) provided a *secA* sequence that clustered separately from the other AY-infected samples. Finally, the sequences assembled from infected canola in Saskatchewan were all identical to the sequence used for probe design, corresponding to the BnAY-TW1 *secA* sequence (Figure 4A).

##### Tuf

The *tuf* gene sequences also provided results that were consistent with expectations. The 16SrX samples yielded sequences that clustered together more tightly than the *secY* sequences, but the different subgroups were readily differentiated (Figure 4B). Again, the ‘*Ca.* P. mali’ sequence was nearly identical to the hybridization target sequence, while the other 16SrX infected samples yielded sequences that were somewhat distinct. The sample infected with ‘*Ca.* P. prunorum’ (ESFY) provided a sequence that was nearly identical to a reference sequence from ESFY. The ‘*Ca.* P. pyri’ samples were identical to one another, and clustered with a short reference sequence for this taxon with a sequence identity of >95%. The 16SrIII and 16SrXII-infected samples also generated sequences that were identical to the respective hybridization target sequence. Unlike *secY*, *tuf* gene sequences were able to differentiate the MBS phytoplasma sequences (Sb7 and Sb41) from the canola samples that were also infected with 16SrI-B, all of which yielded identical sequences. The infected strawberry sample from Quebec (STRAW4) provided a distinct *tuf* sequence from the other 16SrI infected samples. The infected strawberry sample from Mexico, Sb7, generated a second, shorter *tuf* sequence (749 nucleotides) that was unlike any previously reported *tuf* sequence, but shared ~88% sequence identity with 16SrXII-B and 16SrXII-A 284/09 strain.

##### nusA

The *nusA* assemblies tended to be the shortest across all samples analyzed, consistent with the length of the *nusA* gene (1,074 nucleotides, the shortest gene analyzed). These sequences were trimmed to 620 bp, which was the length of the shortest *nusA* assembly (from ESFY). Like *secY* and *tuf*, *nusA* sequences from 16SrX phytoplasmas placed ‘*Ca.* P. pyri’ (PYLR/PD) in a clade with ‘*Ca.* P. prunorum’ (ESFY), with ‘*Ca.* P. mali’ (AP) forming a distinct but related group (Figure 4C). No *nusA* reference sequences are available for ESFY and PYLR/PD. Sequences assembled from 16SrIII and 16SrXII were identical to the hybridization target sequences, as observed with other genes. The *nusA* sequences could differentiate weakly the AY sequences assembled from the infected Mexican strawberries, and all *nusA* sequences assembled from the infected canola were identical to one another and to the target sequence (Figure 4C). STRAW4 clustered separately but within the AY group, as with the other genes (Figure 4C).

## Discussion

The detection, identification, and classification of phytoplasma strains has typically relied upon PCR amplification and sequencing of the 16S rRNA-encoding gene (Zhao et al., 2013). Due to the well-recognized limitations of this approach for various phytoplasma groups, a wide variety of MLST schemes has been described, nearly all of which use PCR amplification and sequencing of specific protein-coding marker genes to improve strain differentiation. An alternative approach to PCR-based MLST was developed and applied to a variety of phytoplasma ribosomal groups, which produces the sequences of seven molecular markers simultaneously and with high accuracy. An important feature of this hybridization-based MLST approach is that it is independent of the design of “universal” primers targeting a subset of phytoplasmas, which overcomes a limitation of some MLST schemes that have been described. In this MLST scheme, hybridization-based gene enrichment was demonstrated to be advantageous. For example, the number of sequencing reads for the BnAY-high sample that mapped to all genes before hybridization was 33, compared to 32,965 reads post-hybridization. These numbers of reads resulted in the assembly of rather long sequences for most of the gene targets (global average of 1929 nucleotides) that are commonly supported by at least hundreds or thousands of reads, providing confidence in the results that are obtained. Reliable reads of this length would be very difficult to obtain using Sanger sequencing from the ends of a clone or amplicon. The assembly of longer protein-coding sequences containing both coding and flanking non-coding regions greatly improves phylogenetic resolution (Gardner et al., 2020). Unlike PCR-based methods, hybridization and assembly does not provide reads with ends that are defined by primer binding locations; therefore, the phylogenetic analysis used fragments that were trimmed manually to the length of the shortest assembly. The ends of the assembled sequence are roughly defined by the sequences of the hybridization probes and are affected by the number of reads mapping to that gene in a given sample. This, in turn, is related to the amount of phytoplasma in the analyzed sample.

Hybridization probe-based detection, differentiation, and classification of difficult-to-culture phytopathogenic bacteria has been previously investigated. For example, in a pioneering study of phytoplasmas, dot and Southern hybridization were used to differentiate phytoplasma strains (Lee et al., 1990, 1992). More recently, enrichment approaches for determining whole-genome sequences of phytoplasmas have been described, using an antibody-based protocol that depletes a sample of eukaryotic DNA (Nijo et al., 2021). An alternative protocol using hybridization probes, similar to that described here, was used to enrich samples infected with the phloem-limited citrus pathogen, ‘*Candidatus* Liberibacter asiaticus’ for target bacterial DNA. This facilitated the assembly of the complete genome of the pathogen, including from samples with low levels (Cq ~30) of the target DNA (Cai et al., 2019).

The results presented here demonstrate that, similar to the observations of (Cai et al., 2019), phytoplasma-infected samples with a low concentration can still generate very long, phylogenetically informative assemblies. Ultimately, a standard fragment length for MLST analysis could be implemented bioinformatically using a tool such as *cutadapt*, which can trim sequences between two specified, degenerate sequences- this tool is used to trim *cpn60* sequences to the universal target by the CpnClassiPhyr (Muirhead et al., 2019a). In almost all cases, for 16S ribosomal and *cpn60* sequences, the hybridization-based MLST approach provided sequences that were of sufficient length and quality for typing using the relevant RFLP-based classifiers, providing additional data that was useful for accurate classification of the samples.

While the hybridization-based approach obviates the need for the design of PCR primers, clearly the phytoplasmas that are targeted are limited to those that possess sequences that are closely related to those of the hybridization probes. The capture of phytoplasma-derived DNA fragments that do not quite match the sequences of the hybridization probes (“off-target” hybridization) is desirable because it can increase the number of distinct phytoplasmas that can be profiled (Ranwez et al., 2011). Some amount of off-target hybridization was observed, consistent with other hybridization-based approaches for determining molecular marker sequences (Gasc and Peyret, 2018). For example, the ribosomal group X-infected samples examined in this work included 16SrX-A, which exactly matches the hybridization target sequence, along with other group X subgroups (16SrX-C and 16SrX-B), which did not exactly match the hybridization probe sequences. In most cases, the lack of a perfect match to the capture probes did not prevent the appearance of the off-target reads in the assembly dataset, since relatively long, robust assemblies were observed for even low concentration samples such as PD. However, *secY* generated off-target assemblies of lesser quality, which is presumably related to the fact that these sequences are more distinct among 16SrX strains. Off-target hybridization was also observed in samples that were infected by phytoplasmas from 16Sr groups that were not represented at all in the hybridization panel, such as Sb7 and Sb41. For example, we observed evidence of a novel *tuf* sequence most likely corresponding to that of 16SrXIII (‘*Ca.* P. hispanicum’), which was found in the Sb7 *tuf* assembly and had no match to anything known at the GenBank database. In a previous study (Pérez-López et al., 2017), sample Sb41 showed evidence of double infection with an on-target (16SrI) and off-target (16SrXIII) phytoplasma. Sample Sb41 had low levels of 16SrXIII and slightly higher levels of 16SrI (Table 3), but only assemblies s corresponding to 16SrI (which typed as closely related to MBS phytoplasma, consistent with their geographic origin in Mexico) were observed. Sample Sb7, however, had much higher levels of 16SrXIII, and was demonstrated to be additionally infected with 16SrI (Table 3), although the levels of the latter were not measured. Nevertheless, assemblies generated from Sb7 mostly corresponded to 16SrI, except for the 16S rRNA gene, which was from 16SrXIII (Table 6). It appears therefore that off-target hybridization does occur, but inefficiently in the case of sequences from phytoplasmas from distinct ribosomal groups (as opposed to subgroups). The protein-coding genes from 16SrXIII also lacked reference genes to use for mapping, which may have resulted in these reads not being represented in the assembly dataset even if they had hybridized to the probes.

Conversely, the capture of non-phytoplasma DNA targets (nonspecific hybridization) is potentially less desirable and can interfere with the assembly of the correct DNA sequences. The number of reads mapping to the different taxonomic marker genes was quite variable across samples and in some cases was quite high, even for samples with relatively low phytoplasma levels (Table 4). This is explained at least in part by variability in read numbers generated in each Illumina run, and variation in the number of samples that were simultaneously processed on a single flowcell (which affects the number of reads allocated to each sample). There may also be technical variability in hybridization stringency and washing efficacy between repeats, which affects the number of reads that are observed in each mapped dataset. The actual number of reads mapping to a given target gene is less important than the quality and length of the assemblies that are produced from these reads. For protein-coding genes such as *cpn60*, virtually all of the mapped reads had very high sequence identity to a probe sequence (Supplementary Figure S1C), representing high-quality sequences that enabled the assembly of long target sequences that were supported by tens to thousands of reads. This contrasts with PCR-based MLST, which typically has two slightly overlapping Sanger reads to support the relatively short contigs that are produced.

In the case of the 16S rRNA gene, many reads were observed in the initial mapping datasets for all phytoplasma-infected samples that corresponded to non-phytoplasma bacteria, along with host chloroplast and mitochondrial genes. In some cases, these reads interfered with the assembly of the correct taxonomic markers, and resulted in samples from uninfected plants showing post-hybridization reads (uninfected grape and canola). The most likely explanation for the preponderance of nonspecific reads in the 16S datasets is that the 16S gene is insufficiently distinct between different bacterial taxa to provide selectivity at both the hybridization and mapping steps. In the case of 16S rRNA genes, many of these “bleed-through” reads corresponded to 16S genes from non-phytoplasma bacteria, as well as 16S-like genes from host chloroplast and mitochondrial genomes. For 16S rRNA target genes only, a second mapping step was required that incorporated these reads; once so cleansed of the nonspecific reads, the assemblies improved considerably. In the case of the small number of protein-coding gene reads that appeared in the uninfected datasets, some amount of nonspecific hybridization and/or mapping can be expected, but these reads did not assemble into the target genes and so may be considered background noise. Therefore, this method is not suited to differentiating phytoplasma positive from negative samples, but that is not its intended use. In most cases, the MLST method will be applied to known positive samples. In addition, the non-hybridized samples showed higher read numbers mapping to all markers, including 16S rRNA, in the samples with lower amounts of phytoplasma (Table 5). While the explanation for this is not obvious, it is worth noting that these relatively small number of reads did not permit the assembly of the complete taxonomic markers in these non-hybridized samples, which demonstrates that necessity of the hybridization step.

This MLST scheme is effective for ribosomal groups 16SrI, 16SrIII, 16SrX, 16SrXII, and various subgroups within each. In addition, this relatively small panel of 351 probes could easily be expanded to design probes to include other phytoplasma groups and subgroups. This process would be straightforward, as it would use already available sequences and software for probe design and follows the hybridization procedures, protocols, and manufacturer guidelines that are well-tested and proven. Another advantage of this method is its potential to analyze a higher number of samples at the same time. For example, by including multiple samples (up to 12) in each hybridization and utilizing a plate format for simultaneous processing of 32 samples, up to 32 × 12 = 384 samples could be processed simultaneously, making high throughput MLST for phytoplasma strains a possibility. Inclusion of other distinct phytoplasmas in the MLST panel is limited only by the availability of reference sequences, as demonstrated by *‘Ca.* P. hispanicum’ (16SrXIII), which would be impossible to represent due to the lack of reference sequences for several genes included in the MLST scheme.

A challenge faced by all molecular methods for differentiating phytoplasmas, including PCR-based methods, is dealing with samples containing either two distinct strains representing a mixed infection, or heterogeneous phytoplasma strains that feature two distinct but closely related phytoplasma 16S rRNA genes. For example, BnAY-TW1 from canola (Town et al., 2018) was found, by PCR and cloning, to contain 2 distinct 16S rRNA-encoding genes, which typed as 16SrI-A and 16SrI-B. Clone sequences generated from the more divergent genes *cpn60* and *rp* were identical to one another, consistent with the presence of a single strain that contains two 16S rRNA genes that type distinctly using RFLP analysis. The genome sequence of strain TW1 was identified as a possible chimeric artifact resulting from the combination of a long-read genome containing 16SrI-A scaffolds, polished with Illumina reads from a 16SrI-B phytoplasma strain (Cho et al., 2020). The hybridization results presented here are inconsistent with this hypothesis, since all the assembled protein-coding genes, and the clone sequences generated, clustered with 16SrI-B sequences. Moreover, the sequences that were assembled were identical or very nearly identical in all cases to the genes identified within the reported TW-1 genome sequence that were used to design the hybridization probes. In addition, the AY-infected strawberry sample from Quebec, STRAW4, provided distinct typing results using the assembled *cpn60* and 16S rRNA genes (Table 6). This sample has also been shown through cloning to contain two distinct 16S genes, which type as 16SrI-R and 16SrI-S along with a single *cpn60* sequence type (Brochu, AS et al., manuscript submitted). In cases of strains with heterogeneous 16S rRNA genes, the hybridization-based method described here would presumably produce a composite sequence from the two distinct loci, much like direct sequencing of PCR-generated amplicon would. Therefore, caution must be used in applying this method to strains that are known to feature 16S rRNA gene heterogeneity, and this should be investigated in each case using PCR amplification, cloning, and sequencing.

In conclusion, this hybridization-based MLST scheme is a method for phytoplasma characterization and provides a proof-of-concept for molecular characterization of other bacterial pathogens that are difficult to culture, despite the limitations listed above. All single-locus classification systems will suffer from a lack of taxonomic resolution due to the limited amount of sequencing information that can be generated from a single marker. This MLST scheme is based on gene targets of proven utility and generates high-quality sequences corresponding to seven different molecular markers. High resolution molecular marker sequences can be determined in this way for phytoplasmas within host plant tissue, even those with low concentrations of this pathogen. Given how straightforward the probe design process is and its high throughput potential, this hybridization-based MLST scheme can be a very efficient molecular tool that provides resolution of closely related phytoplasmas.

The use of DNA sequencing for classification and typing of phytoplasmas will continue to be essential for understanding and monitoring the detrimental effects of phytoplasma infections on crop production. Implementation of a novel, universal, standardized approach for MLST could benefit these efforts and will result in an increased understanding of the spread and effects of these organisms on crop plants worldwide.

## Data availability statement

The datasets generated for this study can be found in GenBank under BioProject accession number 642 PRJNA837572. https://www.ncbi.nlm.nih.gov/bioproject/PRJNA837572.

## Author contributions

HB, TD, DS, and MG were responsible for experimental design. TD, KP-B, DS, and MG performed the experiments. TD and EL analyzed the data. TW and HB provided intellectual input. EL and TD prepared the figures and Supplementary material. TD and KP-B wrote the initial draft of the manuscript. HB, DS, MG, EL, TW, TD, and HB edited the manuscript. TD, TW, and HB acquired the funding, and supervised the project. All authors contributed to the article and approved the submitted version.

## Funding

This work was funded by Canadian Food Inspection Agency grant # SID-P-1802, “Evaluation of Next generation Sequencing (NGS) for the detection and identification of Phytoplasmas.” Graduate stipends for KP-B were provided through two projects funded by Western Grains Research Foundation (Project 1 and 2) and SaskCanola (Project 2), to TW and TD, “An early warning system to predict aster yellows outbreaks in Western Canada: origin and arrival of migrant leafhoppers (AAFC AGR-14988; WGRF # AGR1817), and “Continuing to watch the winds: the origin and arrival of migrant aster leafhoppers and diamondback moths” (AAFC AGR-17913; SaskCanola Ref: CARP ADF2020.409; WGRF Ref: AGR2105.

## Conflict of interest

The authors declare that the research was conducted in the absence of any commercial or financial relationships that could be construed as a potential conflict of interest.

## Publisher’s note

All claims expressed in this article are solely those of the authors and do not necessarily represent those of their affiliated organizations, or those of the publisher, the editors and the reviewers. Any product that may be evaluated in this article, or claim that may be made by its manufacturer, is not guaranteed or endorsed by the publisher.

## Footnotes

^1^ https://www.idtdna.com/site/order/designtool/index/XGENDESIGN
