# Supplementary material for: Multilocus sequence typing of diverse phytoplasmas using hybridization probe-based sequence capture provides high resolution strain differentiation
Source: Front Microbiol. 2022 Sep 29;13:959562. doi: 10.3389/fmicb.2022.959562 (PMC9556853; doi:10.3389/fmicb.2022.959562)
Supplement: Supplementary file 1 [file Table_1.DOCX]

#Phytoplasma multi-gene hyb panel

#new algorithm for mapping and assembly 220122

#step 1: trimmomatic/flash2

#step 2: mapping vs protein coding genes (all types)

#step 3: assemble protein coding genes and determine phytoplasma type - eg cpnclassiphyr

#step 4: mapping vs 16S genes (type-specific) plus mitochondrial/chloroplast and nonspecific bacterial 16S sequences to remove junk

#step 5: assemble 16S genes

#step 6: trim all assemblies to retain only contigs >500 bp

#final product: set of fasta files containing long assemblies for each of 6 protein-coding genes/loci plus 16S

#make conda environment

conda create --name hyb

conda activate hyb

conda install -c bioconda trimmomatic

conda install -c bioconda flash2

#Get read names

mkdir -p /path/to/working/directory/trim

mkdir -p /path/to/working/directory/merge

find /path/to/folder/with/illumina/reads/*.fastq.gz \

-maxdepth 1 \

| sed 's/\/.*\///' \

| sed 's/_R.*$//' \

| sed 's/Undetermined.*$//' \

| sort | uniq \

> /path/to/working/directory/run-id_fastq_files.txt

#step 1A: trimmomatic

#make trimmomatic shell script

nano /path/to/working/directory/trimmomatic.sh

#copy/paste below

#!/bin/bash

#$ -S /bin/bash

#$ -N name_trim

#$ -j y

#$ -cwd

#$ -pe smp 8

source /path/to/home/directory/miniconda3/etc/profile.d/conda.sh

conda activate hyb

for i in `cat /path/to/working/directory/run-id_fastq_files.txt`; do \

trimmomatic PE \

/path/to/folder/with/illumina/reads/$i'_R1_001.fastq.gz' \

/path/to/folder/with/illumina/reads/$i'_R2_001.fastq.gz'\

/path/to/working/directory/trim/$i.paired.fq \

/path/to/working/directory/trim/$i.unpaired.fq \

/path/to/working/directory/trim/$i.paired.reverse.fq \

/path/to/working/directory/trim/$i.unpaired.reverse.fq \

LEADING:3 TRAILING:20 SLIDINGWINDOW:4:15 MINLEN:36 \

; done

#step 1B: flash2 to merge R1, R2

#make merge shell script

nano /path/to/working/directory/merge.sh

#copy/paste below

#!/bin/bash

#$ -S /bin/bash

#$ -N name_merge

#$ -j y

#$ -cwd

#$ -pe smp 4

source /path/to/home/directory//miniconda3/etc/profile.d/conda.sh

conda activate hyb

for i in `cat /path/to/working/directory/run-id_fastq_files.txt` ; do \

flash2 \

-f 550 \

-s 200 \

-r 300 \

/path/to/working/directory/trim/$i'.paired.fq' \

/path/to/working/directory/trim/$i'.paired.reverse.fq' \

-d -d /path/to/working/directory/merge/ \

-o $i.merged.fq \

; done

#step 3: mapping vs protein-coding genes

#Install bowtie2

conda install -c conda-forge tbb

conda install -c bioconda bowtie2

#make bowtie reference files

#Ensure you are using a compute node (qlogin)

#build one ref file for all protein-coding seqs (cpn60; nusA, secY, secA, rp, tuf - call this file ref_protein_seqs.fasta)

#then, build separate ref files containing type-specific 16S genes with junk; eg typeI_16S_junk.fasta

bowtie2-build \

-f /path/to/home/directory/ref_seqs/ref_protein_seqs.fasta \

/path/to/home/directory/ref_seqs/ref_protein_seqs.fasta

bowtie2-build \

-f /path/to/home/directory/ref_seqs/typeI_16S_junk.fasta \

/path/to/home/directory/ref_seqs/typeI_16S_junk.fasta

bowtie2-build \

-f /path/to/home/directory/ref_seqs/typeIII_16S_junk.fasta \

/path/to/home/directory/ref_seqs/typeIII_16S_junk.fasta

bowtie2-build \

-f /path/to/home/directory/ref_seqs/typeX_16S_junk.fasta \

/path/to/home/directory/ref_seqs/typeX_16S_junk.fasta

bowtie2-build \

-f /path/to/home/directory/ref_seqs/typeXII_16S_junk.fasta \

/path/to/home/directory/ref_seqs/typeXII_16S_junk.fasta

#make bowtie (mapping) shell script

nano /path/to/working/directory/map_ref_genes.sh

#copy/paste below

#!/bin/bash

#$ -S /bin/bash

#$ -N name_map

#$ -j y

#$ -cwd

#$ -pe smp 8

source /path/to/home/directory/miniconda3/etc/profile.d/conda.sh

conda activate hyb

#have to do this twice; once for the protein-coding genes using all the samples; once for the type-specific 16S genes plus junk

#for the first round, can do all of the samples at the same time - maps protein-coding genes only for all types

#for the second round, map only those samples containing the relevant types

for i in `cat /path/to/working/directory/run-id_fastq_files.txt` ; do \

bowtie2 --local \

-p 1 \

-x /path/to/home/directory/ref_seqs/typeI_protein_seqs.fasta \

-U /path/to/working/directory/merge/$i'.merged.fq.extendedFrags.fastq' \

-S /path/to/working/directory/$i.ref_genes.sam \

; done

#map vs type-specific 16S genes plus junk. First change filenames list to only those samples containing the relevant type

for i in `cat /path/to/working/directory/run-id_fastq_files.txt` ; do \

bowtie2 --local \

-p 1 \

-x /path/to/home/directory/ref_seqs/typeI_16S_junk.fasta \

-U /path/to/working/directory/merge/$i'.merged.fq.extendedFrags.fastq' \

-S /path/to/working/directory/$i.ref_genes.sam \

; done

#Extract mapped protein-coding or 16S reads for all samples, depending on what has been mapped

#Ensure you are using a compute node (qlogin)

for i in `cat /path/to/working/directory/run-id_fastq_files.txt` ; do \

samtools view -bS \

/path/to/working/directory/$i'.ref_genes.sam' \

> /path/to/working/directory/$i.ref_genes.bam \

; done

for i in `cat /path/to/working/directory/run-id_fastq_files.txt` ; do \

samtools sort \

/path/to/working/directory/$i'.ref_genes.sam' \

> /path/to/working/directory/$i.ref_genes.bam.sorted \

; done

for i in `cat /path/to/working/directory/run-id_fastq_files.txt` ; do \

samtools index \

/path/to/working/directory/$i'.ref_genes.bam.sorted' \

; done

#Extract mapped reads and corresponding fastq for each protein-coding gene

#can submit as a job to do all at once

nano /path/to/working/directory/mapped_fastq/mapped_fastq.sh

#copy/paste below

#!/bin/bash

#$ -S /bin/bash

#$ -N name_map_typeI

#$ -j y

#$ -cwd

#$ -pe smp 1

source /path/to/home/directory/miniconda3/etc/profile.d/conda.sh

conda activate hyb

#make sure gene names below (eg "secY_AY-I" PERFECTLY matches the corresponding gene name in ref_genes.fasta (after, ">") - or you will get this error: region "secY" specifies an unknown reference name. Continue anyway. And no reads will match. So, need to extract reads vs 1 group at a time - eg secY_AY-I

#secY

for i in `cat /path/to/working/directory/run-id_fastq_files.txt` ; do \

samtools view -bh \

/path/to/working/directory/$i'.ref_genes.bam.sorted' \

secY_AY-I \

> /path/to/working/directory/$i.mapped.secY.bam \

; done

for i in `cat //path/to/working/directory/run-id_fastq_files.txt` ; do \

samtools fastq \

-0 /path/to/working/directory/mapped_fastq/$i.mapped.secY.fastq \

/path/to/working/directory/$i'.mapped.secY.bam' \

; done

#cpn60 - skip for type III (no cpn60)

for i in `cat /path/to/working/directory/run-id_fastq_files.txt` ; do \

samtools view -bh \

/path/to/working/directory/$i'.ref_genes.bam.sorted' \

cpn60_AY-I \

> /path/to/working/directory/$i.mapped.cpn60.bam \

; done

for i in `cat /path/to/working/directory/run-id_fastq_files.txt` ; do \

samtools fastq \

-0 /path/to/working/directory/mapped_fastq/$i.mapped.cpn60.fastq \

/path/to/working/directory/$i'.mapped.cpn60.bam' \

; done

#tuf

for i in `cat /path/to/working/directory/run-id_fastq_files.txt` ; do \

samtools view -bh \

/path/to/working/directory/$i'.ref_genes.bam.sorted' \

tuf_AY-I \

> /path/to/working/directory/$i.mapped.tuf.bam \

; done

for i in `cat /path/to/working/directory/run-id_fastq_files.txt` ; do \

samtools fastq \

-0 /path/to/working/directory/mapped_fastq/$i.mapped.tuf.fastq \

/path/to/working/directory/$i'.mapped.tuf.bam' \

; done

#rp

for i in `cat /path/to/working/directory/run-id_fastq_files.txt` ; do \

samtools view -bh \

/path/to/working/directory/$i'.ref_genes.bam.sorted' \

rp_AY-I \

> /path/to/working/directory/$i.mapped.rp.bam \

; done

for i in `cat /path/to/working/directory/run-id_fastq_files.txt` ; do \

samtools fastq \

-0 /path/to/working/directory/mapped_fastq/$i.mapped.rp.fastq \

/path/to/working/directory/$i'.mapped.rp.bam' \

; done

#nusA

for i in `cat /path/to/working/directory/run-id_fastq_files.txt` ; do \

samtools view -bh \

/path/to/working/directory/$i'.ref_genes.bam.sorted' \

nusA_AY-I \

> /path/to/working/directory/$i.mapped.nusA.bam \

; done

for i in `cat /path/to/working/directory/run-id_fastq_files.txt` ; do \

samtools fastq \

-0 /path/to/working/directory/mapped_fastq/$i.mapped.nusA.fastq \

/path/to/working/directory/$i'.mapped.nusA.bam' \

; done

#secA

for i in `cat /path/to/working/directory/run-id_fastq_files.txt` ; do \

samtools view -bh \

/path/to/working/directory/$i'.ref_genes.bam.sorted' \

secA_AY-I \

> /path/to/working/directory/$i.mapped.secA.bam \

; done

for i in `cat /path/to/working/directory/run-id_fastq_files.txt` ; do \

samtools fastq \

-0 /path/to/working/directory/mapped_fastq/$i.mapped.secA.fastq \

/path/to/working/directory/$i'.mapped.secA.bam' \

; done

#16S genes; after doing mapping vs type-specific ref seqs plus junk

for i in `cat /path/to/working/directory/run-id_fastq_files.txt` ; do \

samtools view -bh \

/path/to/working/directory/$i'.ref_genes.bam.sorted' \

16S_AY-I \

> /path/to/working/directory/$i.mapped.16S.bam \

; done

for i in `cat /path/to/working/directory/run-id_fastq_files.txt` ; do \

samtools fastq \

-0 /path/to/working/directory/mapped_fastq/$i.mapped.16S_AY-I.fastq \

/path/to/working/directory/$i'.mapped.16S.bam' \

; done

##################################

#How many reads mapped to each gene?

grep -c M01666 /path/to/working/directory/all-mapped_fastq/*.16S_AY-I.fastq

#Assembly

mkdir ~/software/

cd ~/software

wget https://www.bcgsc.ca/platform/bioinfo/software/trans-abyss/releases/2.0.1/transabyss-2.0.1.zip

unzip transabyss-2.0.1.zip

#modify .bashrc file

cd ~

nano .bashrc

#add the following:

#added by townj

export PATH="/home/AAFC-AAC/dumonceauxt/software/transabyss-2.0.1/:$PATH"

#log off of server, then log back on

#Add igraph to conda environment

conda activate hyb

conda install -c conda-forge python-igraph

#Assemble

#make sure you are using qlogin session

mkdir -p /path/to/working/directory/assemblies

#cpn60

for i in `cat /path/to/working/directory/run-id_fastq_files.txt` ; do \

transabyss --se \

/path/to/working/directory/mapped_fastq/$i'.mapped.cpn60.fastq' \

-k 32 \

--outdir /path/to/working/directory/assemblies/$i'_cpn60' \

; done

#secA

for i in `cat /path/to/working/directory/run-id_fastq_files.txt` ; do \

transabyss --se \

/path/to/working/directory/mapped_fastq/$i'.mapped.secA.fastq' \

-k 32 \

--outdir /path/to/working/directory/assemblies/$i'_secA' \

; done

#secY

for i in `cat /path/to/working/directory/run-id_fastq_files.txt` ; do \

transabyss --se \

/path/to/working/directory/mapped_fastq/$i'.mapped.secY.fastq' \

-k 32 \

--outdir /path/to/working/directory/assemblies/$i'_secY' \

; done

#tuf

for i in `cat /path/to/working/directory/run-id_fastq_files.txt` ; do \

transabyss --se \

/path/to/working/directory/mapped_fastq/$i'.mapped.tuf.fastq' \

-k 32 \

--outdir /path/to/working/directory/assemblies/$i'_tuf' \

; done

#nusA

for i in `cat /path/to/working/directory/run-id_fastq_files.txt` ; do \

transabyss --se \

/path/to/working/directory/mapped_fastq/$i'.mapped.nusA.fastq' \

-k 32 \

--outdir /path/to/working/directory/assemblies/$i'_nusA' \

; done

#rp

for i in `cat /path/to/working/directory/run-id_fastq_files.txt` ; do \

transabyss --se \

/path/to/working/directory/mapped_fastq/$i'.mapped.rp.fastq' \

-k 32 \

--outdir /path/to/working/directory/assemblies/$i'_rp' \

; done

#16S

for i in `cat /path/to/working/directory/run-id_fastq_files.txt` ; do \

transabyss --se \

/path/to/working/directory/mapped_fastq/$i'.mapped.16S_AY-I.fastq' \

-k 32 \

--outdir /path/to/working/directory/assemblies/$i'_16S'\

; done

#step6 - trim assemblies to retain only those >500 bp (code added by Kevin Muirhead)

#if miniconda not there, go get it

wget https://repo.anaconda.com/miniconda/Miniconda3-latest-Linux-x86_64.sh

sh Miniconda3-latest-Linux-x86_64.sh

#... follow miniconda3 installation directions... Mostly pressing enter. Installs in home by default

conda create --name bioawk_env

conda activate bioawk_env

conda install -c bioconda bioawk

#this command will now trim transabyss-final.fa to only those with a length >=500 bp and output it to test-seq-l500.fa. Can change names or length as needed.

bioawk -c fastx 'length($seq) >= 500{ print ">"$name" "$comment; print $seq }' transabyss-final.fa > test-seq-l500.fa
