# Supplementary material for: Multilocus sequence typing of diverse phytoplasmas using hybridization probe-based sequence capture provides high resolution strain differentiation
Source: Front Microbiol. 2022 Sep 29;13:959562. doi: 10.3389/fmicb.2022.959562 (PMC9556853; doi:10.3389/fmicb.2022.959562)
Supplement: Supplementary file 4 [file Data_Sheet_2.DOCX]

Multilocus sequence typing of diverse phytoplasmas using hybridization probe-based sequence capture provides high resolution strain differentiation

Karolina Pusz-Bochenska ^1,2^, Edel Perez-Lopez ^3, 4^, Tyler J. Wist^1,2^, Harvinder Bennypaul^5^, Daniel Sanderson^5^, Margaret Green ^5^, Tim J. Dumonceaux^1,6*^

^1^Agriculture and Agri-Food Canada Saskatoon Research and Development Centre, Saskatoon, Saskatchewan, Canada

^2^Department of Biology, University of Saskatchewan, Saskatoon, Saskatchewan, Canada

^3^ Centre de recherche et d'innovation sur les végétaux (CRIV), Faculté des sciences de l'agriculture et de l'alimentation, Département de phytologie, Université Laval, Québec, Québec, Canada

^4^Institut de Biologie Intégrative et des systèmes, Université Laval, Québec, Canada.

^5^CFIA Centre for Plant Health, 8801 East Saanich Road, North Saanich, BC

^6^Department of Veterinary Microbiology, University of Saskatchewan, Saskatoon, Saskatchewan, Canada

*** Correspondence:**Tim J. Dumonceaux
tim.dumonceaux@agr.gc.ca

Supplementary Material

| phytoplasma target | gene target | primer sequences (5'-3') | TNA probe sequence (5'-3') (5'FAM; 3' BHQ-1) | amplification conditions |
| --- | --- | --- | --- | --- |
| *'Ca.* P. asteris’ (16SrI) (Olivier et al., 2017) | *cpn60* | TGGAGTTATTAATGTTGATG | ATCCTTCAACAACTTCTAATTCTG | 95°C, 30 sec (1x); 95°C-30 sec, 59°C-30 sec, 72°C, 30 sec (data collection) (40x) |
|  |  | GGAGAAGCATATCCTTTA |  |  |
| *'Ca.* P. prunorum’ ESFY (16SrX-F) | *cpn60* | GTCAGTTGAATTAGAACATC | AACTTCATTTTCTACAGAATCAGCAA | 95°C, 30 sec (1x); 95°C-10 sec, 57°C-10 sec, 72°C, 20 sec (data collection) (40x) |
|  |  | GTTCCTTTCATTTTATTGATA |  |  |
| *'Ca.* P. pyri’ PYLR-PD (16SrX-C) | *cpn60* | GACGAATCAAAAGGATTTG | TGACAAAGGTTATGCTTCGCC | 95°C, 30 sec (1x); 95°C-10 sec, 57°C-10 sec, 72°C, 20 sec (data collection) (40x) |
|  |  | ACCTTGTTTCACTACTTCTTCT |  |  |
| *'Ca.* P. mali’ (AP) (16SrX-A) | *cpn60* | TGGTGTTATTAACGTTGA | CGACTGACATAGTTTCCCTATTG | 95°C, 30 sec (1x); 95°C-10 sec, 57°C-10 sec, 72°C, 20 sec (data collection) (40x) |
|  |  | TGGTCTGTTACTAAAACTA |  |  |
| '*Ca.* P. pruni’ (16SrIII) (Christensen et al., 2004) | 16S | CGTACGCAAGTATGAAACTTAAAGGA | TGAcGGgAcTCCGcA (3' IowaBlack FQ) locked nucleic acid bases are indicated in lower case | 95°C, 5 min (1x); 95°C, 15 sec; 64°C, 1 min (40x) (data collection) |
|  |  | TCTTCGAATTAAACAACATGATCCA |  |  |
| '*Ca*. P. solani’ (16SrXII) | *cpn60* | TCCGACAGAGAAAGTATG | TTTGATTCTTTGACCACTCCTTCTAAA | 95°C, 30 sec (1x); 95°C-10 sec, 57°C-10 sec, 72°C, 20 sec (data collection) (40x) |
|  |  | TCAGCGATGATCAATAAAG |  |  |

**Table S1**. Primer and probe sequences, along with amplification conditions, used for qPCR assays.

|  | | first map | | | second map | | |
| --- | --- | --- | --- | --- | --- | --- | --- |
| Sample | Group | raw reads | read proportion | longest assembled contig | raw reads | read proportion | longest assembled contig |
| BnAY-high | 16SrI-A/B | 7636 | 0.163 | 1488 | 8402 | 0.179 | 2654 |
| BnAY-medium | 16SrI-A/B | 22410 | 0.235 | 1168 | 11399 | 0.119 | 2557 |
| BnAY-low | 16SrI-A/B | 22026 | 0.246 | 0 (chloroplast sequence) | 1134 | 0.013 | 2230 |
| BnAY-vlow | 16SrI-A/B | 27508 | 0.256 | 0 (chloroplast sequence) | 285 | 0.003 | 925 |
| BnH-0 | -- | 23984 | 0.243 | 0 (chloroplast sequence) | 192 | 0.002 | 813 |
| ‘*Ca.* P prunorum’ (ESFY) | 16SrX-F | 7982 | 0.069 | 1317 | 5000 | 0.043 | 2184 |
| ‘*Ca.* P mali’ (AP) | 16SrX-A | 11121 | 0.091 | 2287 | 9917 | 0.081 | 2280 |
| ‘*Ca.* P pyri’ (PD) | 16SrX-C | 5180 | 0.060 | 1305 | 729 | 0.008 | 2085 |
| ‘*Ca.* P pyri’ (PYLR) | 16SrX-C | 17421 | 0.151 | 1055 | 10033 | 0.087 | 2218 |
| ‘*Ca.* P pruni’ – 2A1 | 16SrIII-A | 6809 | 0.070 | 2435 | 6225 | 0.064 | ---^1^ |
| ‘*Ca.* P pruni’ – 6A1 | 16SrIII-A | 6434 | 0.078 | 2400 | 6150 | 0.074 | --- |
| ^1^reads not assembled because a contig of sufficient length was generated without the 2-step strategy | | | | | | | |

**Table S2.** 16S mapping results and clean up using a two-step mapping strategy.


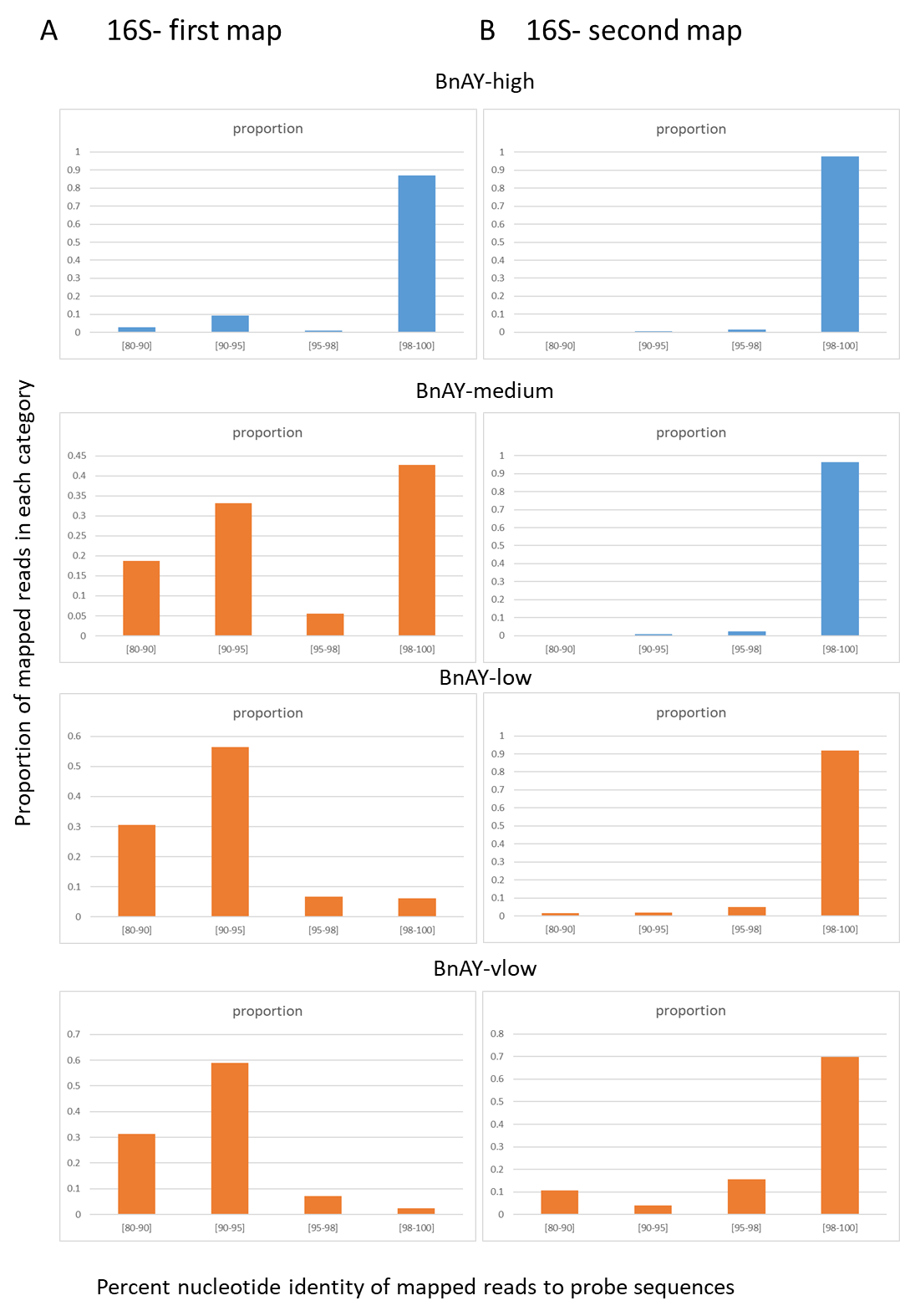


**
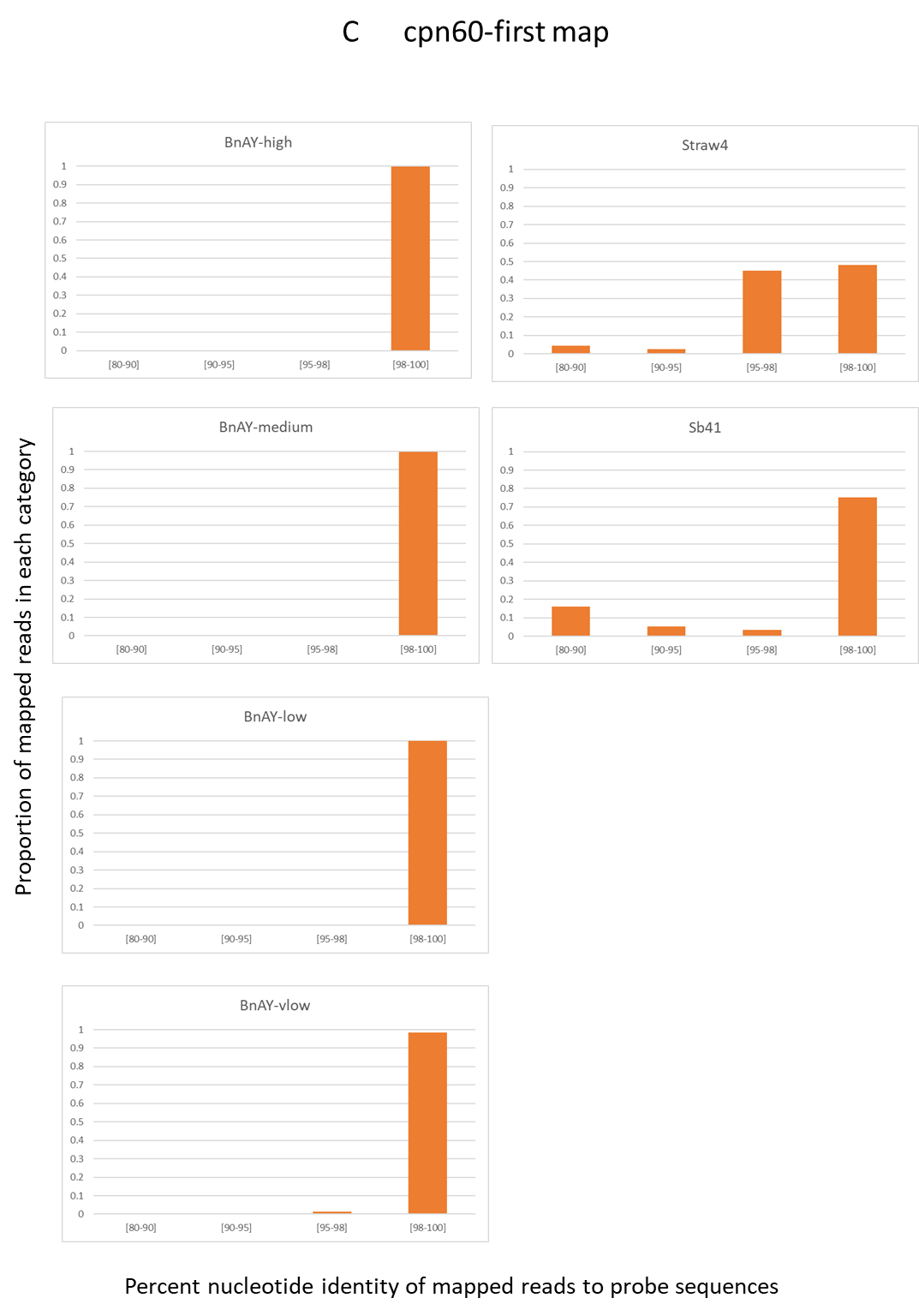
**

**Supplementary Figure S1.** BLAST results of post-hybridization mapped reads at each spike level compared to hybridization probe sequences. The proportions of mapped reads that matched one of the probe sequences at various percent identity ranges are shown. A. Initial mapping results, which did not yield suitable assemblies due to the number of nonspecific reads in the assembly dataset (Table S2) B. Results after implementing a second mapping step that used nonspecific assemblies from the initial mapping step (other bacterial 16S genes, mitochondrial, chloroplast genes) as mapping targets. This significantly improved the number of reads that closely matched one of the probe sequences, and resulted in improved assemblies at all spike levels (Table S2). C. Mapping results for *cpn60* sequences. A single round of mapping against the target gene yielded nearly all reads with a very close match to a probe sequence, resulting in high quality assemblies at all spike levels (Table 6).


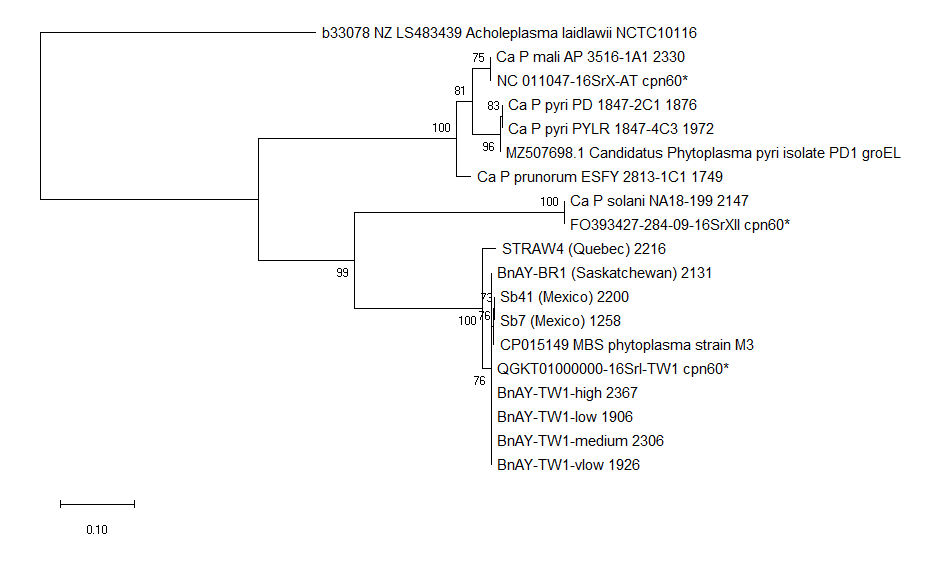


**Supplementary Figure S2.** Phylogenetic analysis of *cpn60* sequences generated using the hybridization-based MLST method. Sequences were trimmed manually to the length of the shortest available sequence (~1175 bp) prior to phylogenetic analysis using the neighbour-joining method as described in Methods. Sequences that were assembled from hybridized samples are labelled according to Table 3. Sequences used for probe design are labelled with an asterisk (*).

References

Christensen, N.M., Nicolaisen, M., Hansen, M., and Schulz, A. (2004). Distribution of Phytoplasmas in Infected Plants as Revealed by Real-Time PCR and Bioimaging. 17(11)**,** 1175-1184. doi: 10.1094/mpmi.2004.17.11.1175.

Olivier, C., Dumonceaux, T., Perez-Lopez, E., Wist, T., Elliott, B., and Vail, S. (2017). "Detection, symptomatology and management of Aster yellows disease in canola," in *Integrated Management of Insect Pests on Canola and Other Brassica Oilseed Crops,* ed. G.V.P. Reddy. CABI).
